# Supplementary material for: Photothermal recycling of waste polyolefin plastics into liquid fuels with high selectivity under solvent-free conditions
Source: Nat Commun. 2023 Jul 15;14:4242. doi: 10.1038/s41467-023-40005-6 (PMC10349850; doi:10.1038/s41467-023-40005-6)
Supplement: Supplementary file 1 — Supplementary Information [file 41467_2023_40005_MOESM1_ESM.pdf]

Supplementary Information

Photothermal recycling of waste polyolefin plastics into liquid fuels with high selectivity under solvent-free conditions

Yingxuan Miao[1, 2], Yunxuan Zhao[1, *], Geoffrey I.N. Waterhouse[3], Run Shi[1], Li-Zhu Wu[1] and Tierui Zhang[1, 2, *]

[1] Key Laboratory of Photochemical Conversion and Optoelectronic Materials, Technical Institute of Physics and Chemistry, Chinese Academy of Sciences, Beijing 100190, China

[2] Center of Materials Science and Optoelectronics Engineering, University of Chinese Academy of Sciences, Beijing 100049, China

[3] School of Chemical Sciences, The University of Auckland, Auckland 1142, New Zealand

* Correspondence and requests for materials should be addressed to Y. Z. (yunxuan@mail.ipc.ac.cn) or T. Z. (tierui@mail.ipc.ac.cn)

Supplementary Materials

Deuterated chloroform ($CDCl_3$), dichloromethane (CH_2Cl_2) and mesitylene were obtained from Aladdin. Cyclohexane (HPLC-grade) and ultra-high-molecular-weight polyethylene (UHMWPE) were purchased from Macklin. High-density polyethylene (HDPE) was obtained from Sigma Aldrich. Low-density polyethylene (LDPE) and Ru powder were obtained from Alfa Aesar. LDPE bags (Aladdin, C4722-11) or polypropylene granules (Aladdin) were frozen in liquid nitrogen, and then pulverized into millimeter-sized pieces. n-hexadecane was purchased from Innochem. Di-tert-butylhydroxytoluene (BHT) was purchased from Acros. 1,2,4-trichlorobenzene (TCB) was purchased from Honeywell. P25-TiO_2 was purchased from Degussa AG. The H_2/Ar (v/v = 10/90), H_2/Ar (v/v = 30/70), and H_2/N_2 (v/v = 70/30) gas mixtures were purchased from Beijing Haipu Gas Co., Ltd. All reagents were of analytical grade and used as received without further purification.

Supplementary Methods

Material characterizations. X-ray diffraction (XRD) patterns of catalyst powders were collected on a Bruker D8 Focus X-ray diffractometer (Cu Kα radiation source, 40 kV, λ = 1.5405 Å). Transmission electron microscopy (TEM) and high-resolution TEM (HRTEM) images were taken on a JEOL-2100F microscope operating at an accelerating voltage of 200 kV. The instrument was also equipped with high-angle annular dark-field scanning TEM (HAADF-STEM) and EDX elemental mapping capabilities. Attenuated total reflectance infrared spectroscopy (Bruker, VERTEX 70v) data were collected over the wavenumber range 400-4000 cm^{-1} at a resolution of 1 cm^{-1}. 64 scans were accumulated for each spectrum. Ultraviolet-visible (UV-Vis) diffuse reflectance spectra were collected over the wavelength range 200-2500 nm on a Cary 7000 (U.S Varian) spectrometer equipped with an integrating sphere attachment.

Thermogravimetric Analysis (TGA) and differential scanning calorimetry (DSC). TGA curves for LDPE were collected on an EXSTAR instrument. Samples were heated from 25 °C to 800 °C at 10 °C/min in an argon flow. DSC curves for pristine LDPE and polymeric residues were collected on a DSC 200 PC (NETZSCH, Germany). Samples were heated from 60 °C to 160 °C at 10 °C/min under flowing nitrogen (50 mL/min).

Measurements were performed in triplicate to ensure consistency of the results.

Measurement of molecular weight. The weight-average molecular weight (M_w), number-average molecular weight (M_n), and molecular weight distributions ($Đ = M_w/M_n$) of the different polymers were determined by high-temperature gel permeation chromatography (GPC) on an Agilent PL-GPC-220 instrument equipped with three detectors (a two-angle laser light scattering detector, a refractive index detector, and a viscosity detector) and three PL-Gel Mixed B columns. The samples were dissolved in TCB containing 0.01 wt.% BHT and heated at 150 °C for at least 2 h. Elution of columns used a 1 mL/min flow of TCB (with BHT) at 150 °C. Before the sample measurements, the GPC system was calibrated using monomodal, linear polyethylene standards (Varian).

Gas chromatography. Gaseous products of the photothermal polyolefin conversion experiments were analyzed on a GC-2014C gas chromatograph (Shimadzu Co., Japan) equipped with three channels. The first channel analyzed hydrocarbons (C_1-C_7) using a HP PLOT Al_2O_3 column with He as the carrier gas and a flame ionization detector (FID). The second channel analyzed CO_2, N_2, Ar, O_2, CH_4, and CO with a combination of micropacket Haysep Q, H-N, and Molsieve 13× columns using He as the carrier gas and a thermal conductivity detector (TCD). The third channel analyzed H_2 using a micropacket HayeSep Q and Molsieve 5 Å column with N_2 as the carrier gas and a TCD detector. The gaseous products were quantified by an external standard method based on methane. A Shimadzu GC-2014 gas chromatograph (Shimadzu Co., Japan) equipped with a HP-5 column and FID with N_2 as carrier was used for the analysis of higher hydrocarbons (C_5-C_{37}).

Calculation of polyolefin degradation percentage

The polyolefin degradation percentage during ambient-pressure photothermal experiments was calculated using the equation:

$$\text{Degradation percentage } (R_d)[\%] = \frac{M_i - M_r}{M_s} \times 100$$

Where M_i is the input mass of Ru/TiO_2 and polyolefin substrate, M_r is the mass of Ru/TiO_2 and unreacted polymer residue, M_s is the input mass of polyolefin substrate.

Standard deviation calculated from two samples was utilized in the error bar.

Analysis of liquid/waxy products. ^1H nuclear magnetic resonance (^1H NMR) analysis of liquid/waxy products was conducted in $CDCl_3$ at 25 °C using a Bruker Avance DPX 600 MHz spectrometer. High-temperature gas chromatography (HTGC) was performed on an AC Agilent-6890 equipped with D7169 capillary column (5 m × 0.53 mm × 0.09 μm) using the NB/SH/T 0829-2010 standard method.

3D-FDTD stimulation. The local surface plasmon resonance (LSPR) of Ru nanoparticles was stimulated by the 3-dimensional finite-difference time-domain (3D-FDTD) method so that electric field distribution could be visualized. 3D FDTD simulations were used to determine the near-field intensities around the Ru nanoparticles loaded over the TiO_2 substrate. Electromagnetic field distribution was calculated for ruthenium nanoparticles with diameters of 50 nm, 10 nm, and 2 nm on TiO_2. A plane wave polarized light of wavelength 200-800 nm was used along the z-axis. The mesh override region was set to 0.1 nm and the overall simulation time limited to 500 fs.

Supplementary Figures

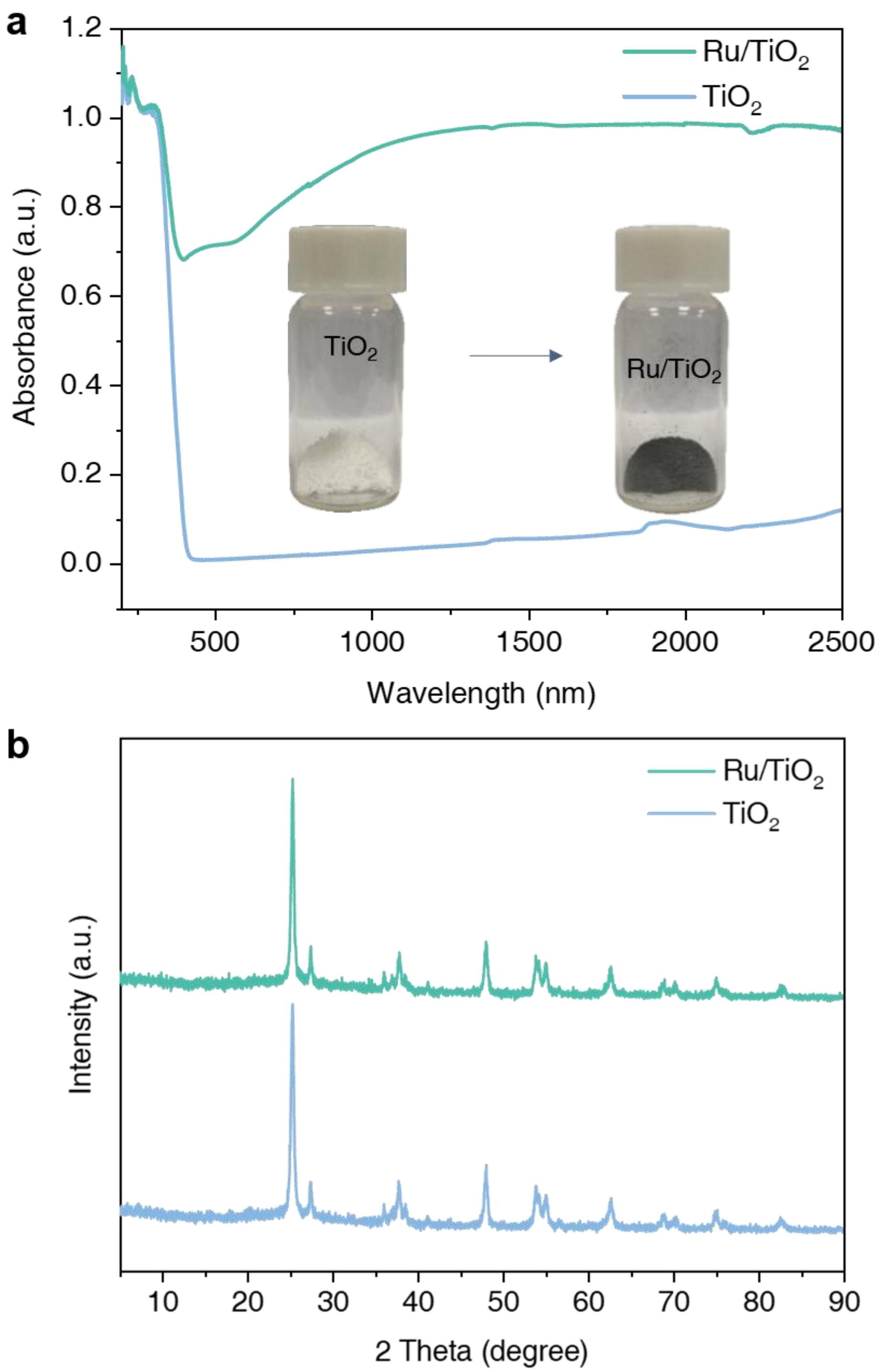

Supplementary Fig. 1 | Light absorption and crystal phases of Ru/TiO_2. a, UV-Vis diffuse reflectance spectra of TiO_2 and Ru/TiO_2, with corresponding photographs of the samples. **b**, XRD patterns of TiO_2 and Ru/TiO_2.

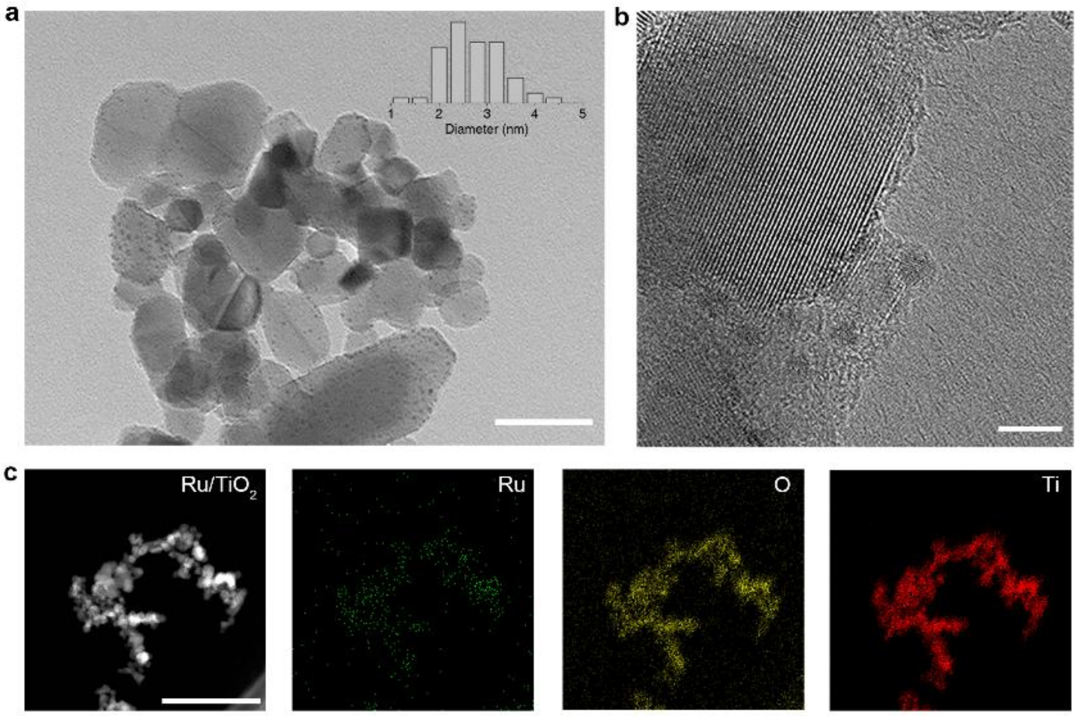

Supplementary Fig. 2 | Electron micrographs of Ru/TiO_2. a, TEM image of Ru/TiO_2, scale bar, 50 nm. Inset: corresponding particle size distribution of Ru nanoparticles on Ru/TiO_2. **b**, HRTEM image of Ru/TiO_2. Scale bar, 5 nm. **c**, Element maps showing the uniform distribution of Ru, O, and Ti in Ru/TiO_2. Scale bar, 300 nm.

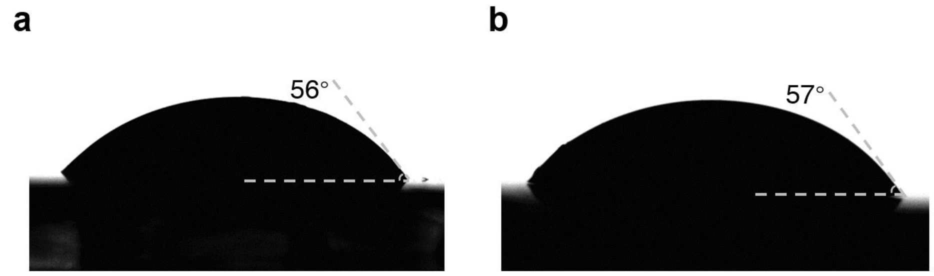

Supplementary Fig. 3 | Contact angles of LDPE droplet on Ru/TiO_2 with light irradiation. LDPE droplet contact angles on a glass substrate coated with Ru/TiO_2 under **a**, Xe lamp irradiation and **b**, UV irradiation at 300 °C in an argon atmosphere.

A small decrease of static contact angle of LDPE droplets under Xe lamp (*i.e.*, photothermal condition, θ = 56°) or UV irradiation (θ = 57°) at 300 °C compared to data that without light irradiation (*i.e.*, thermal condition, θ = 60°). The slightly decreased contact angle may result from elevated mobility or reorientation of side chains or pendant groups on the macromolecules under UV irradiation[1,2].

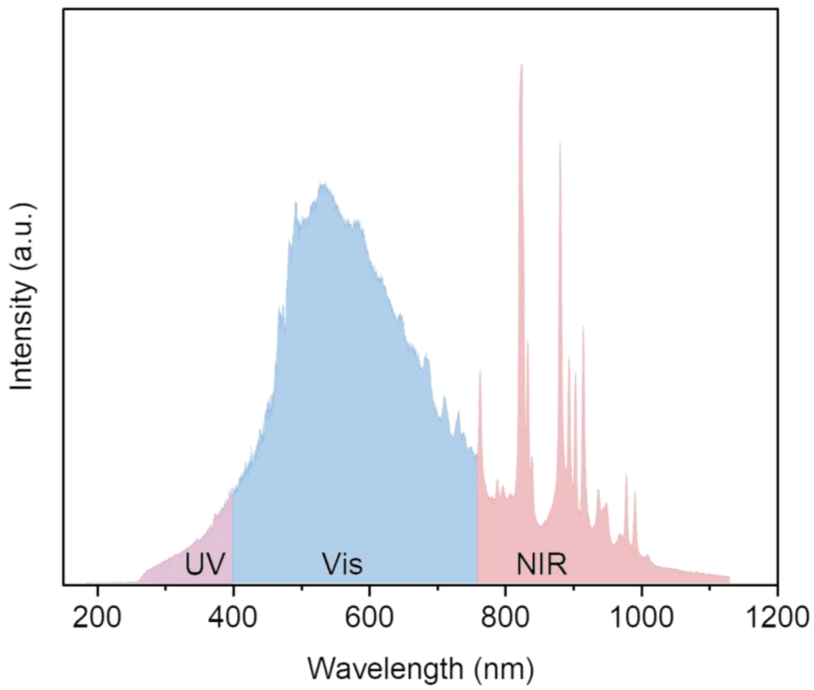

Supplementary Fig. 4 | Emission spectrum of the Xe lamp.

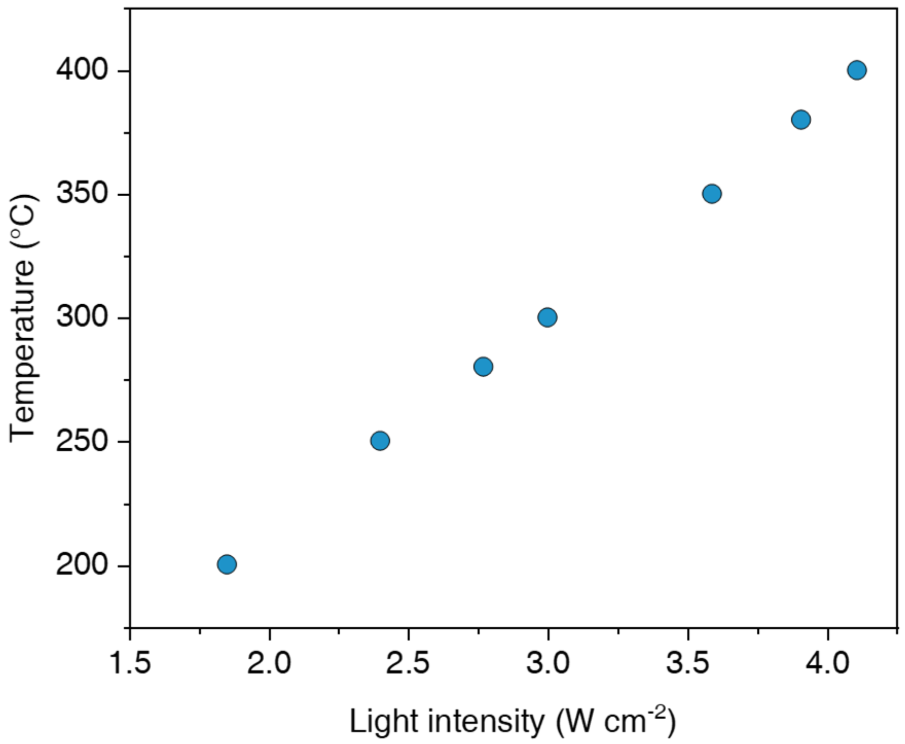

Supplementary Fig. 5 | Temperature at different light intensities. The temperature of a mixture of LDPE and Ru/TiO_2 catalyst under Xe lamp irradiation varied depending on the light intensity. The temperature was monitored by a K-type thermocouple in direct contact with the mixture of LDPE and Ru/TiO_2 catalyst. Temperatures at specific light intensities were: 200 °C at 1.85 W cm^{-2}, 250 °C at 2.40 W cm^{-2}, 280 °C at 2.77 W cm^{-2}, 300 °C at 3.00 W cm^{-2}, 350 °C at 3.59 W cm^{-2}, 380 °C at 3.91 W cm^{-2}, and 400 °C at 4.11 W cm^{-2}.

The temperature of the LDPE and Ru/TiO_2 catalyst mixture showed a linear correlation with the light intensity of the Xe lamp, thus allowing accurate control of the photothermal reaction temperature by adjustment of the light intensity. In addition, we could also adjust the reaction temperature by auxiliary heating with a heating element or cooling using circulating water.

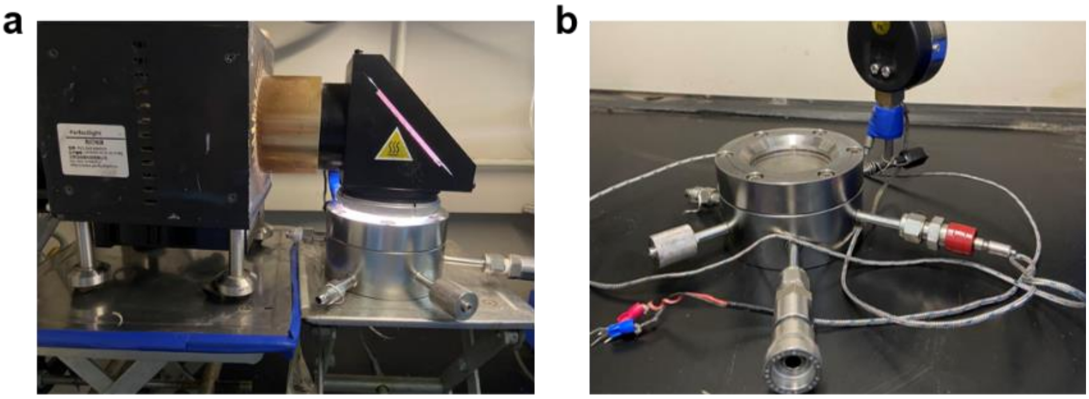

Supplementary Fig. 6 | Digital images of the ambient-pressure photothermal stainless reactor with quartz window. **a**, The reactor during ambient-pressure photothermal polyolefin recycling experiments under Xe lamp irradiation. **b**, The reactor showing a pressure gauge, gas inlet and outlet ports, and thermocouple for temperature monitoring.

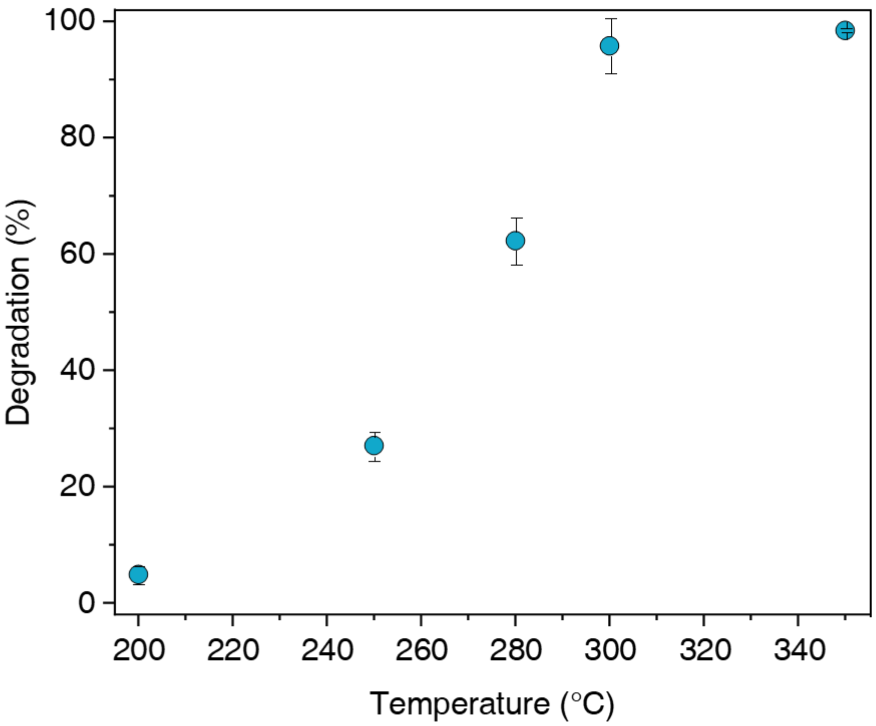

Supplementary Fig. 7 | Degradation percentage of LDPE at different photothermal reaction temperatures over Ru/TiO_2. Reaction conditions: 1 bar H_2/Ar (v/v = 30/70), 80 mg LDPE, 20 mg Ru/TiO_2, reaction time 20 h.

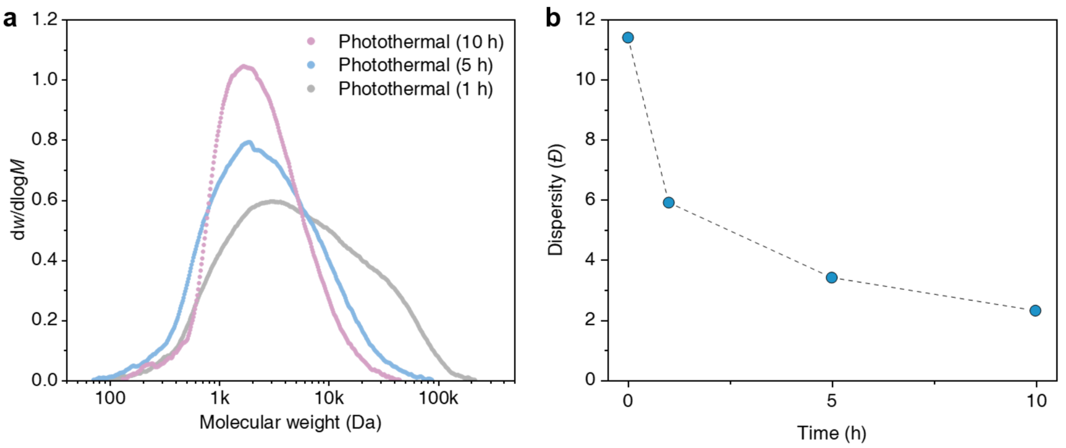

Supplementary Fig. 8 | GPC analysis of polymer residues at different photothermal reaction times. a, Molecular weight distribution and **b**, Dispersity (*Đ*) plots of the polymer residues at different photothermal reaction times. $M_{w\text{-}1h}$ = 12.9 kDa, $M_{w\text{-}5h}$ = 4.7 kDa, $M_{w\text{-}10h}$ = 3.4 kDa. Corresponding dispersities were $Đ_{1h}$ = 5.9, $Đ_{5h}$ = 3.4, and $Đ_{10h}$ = 2.3, respectively. Reaction conditions: 1 bar H_2/Ar (v/v = 30/70), 300 °C (provided by 3.0 W cm^{-2} Xe lamp), 80 mg LDPE, 20 mg Ru/TiO_2.

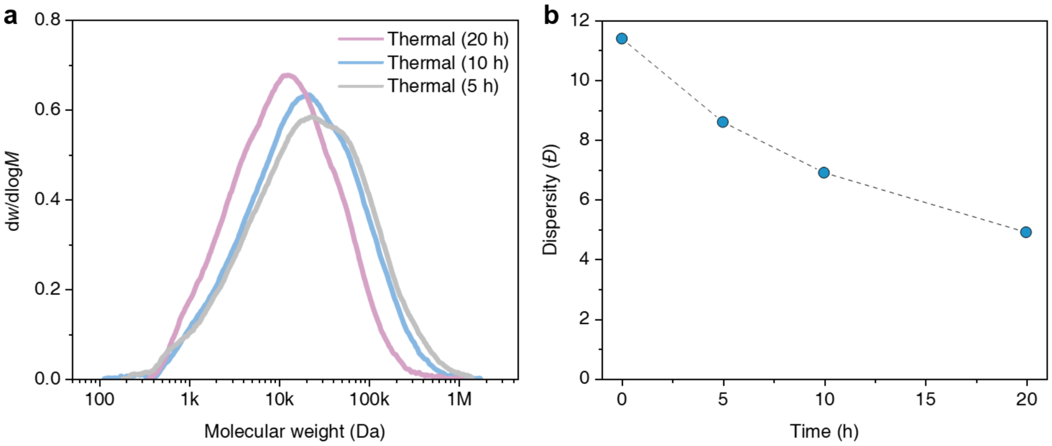

Supplementary Fig. 9 | GPC analysis of polymer residues at different thermal reaction times. a, Molecular weight distribution and **b**, Dispersity ($Đ$) plots of the polymer residues at different thermal reaction times. $M_{w\text{-}5h}$ = 57.3 kDa, $M_{w\text{-}10h}$ = 46.1 kDa, $M_{w\text{-}20h}$ = 25.0 kDa. Corresponding dispersities were $Đ_{5h}$ = 8.6, $Đ_{10h}$ = 6.9, and $Đ_{20h}$ = 4.9, respectively. Reaction conditions: 1 bar H_2/Ar (v/v = 30/70), 300 °C (provided by heating element), 80 mg LDPE, 20 mg Ru/TiO_2.

As shown in Supplementary Fig. 9, thermal degradation of LDPE over Ru/TiO_2 results in a wide dispersity ($Đ = M_w/M_n$). This is attributed to the random scission of C-C bonds and insufficient cracking of the high-molecular-weight portion of polymer residues.

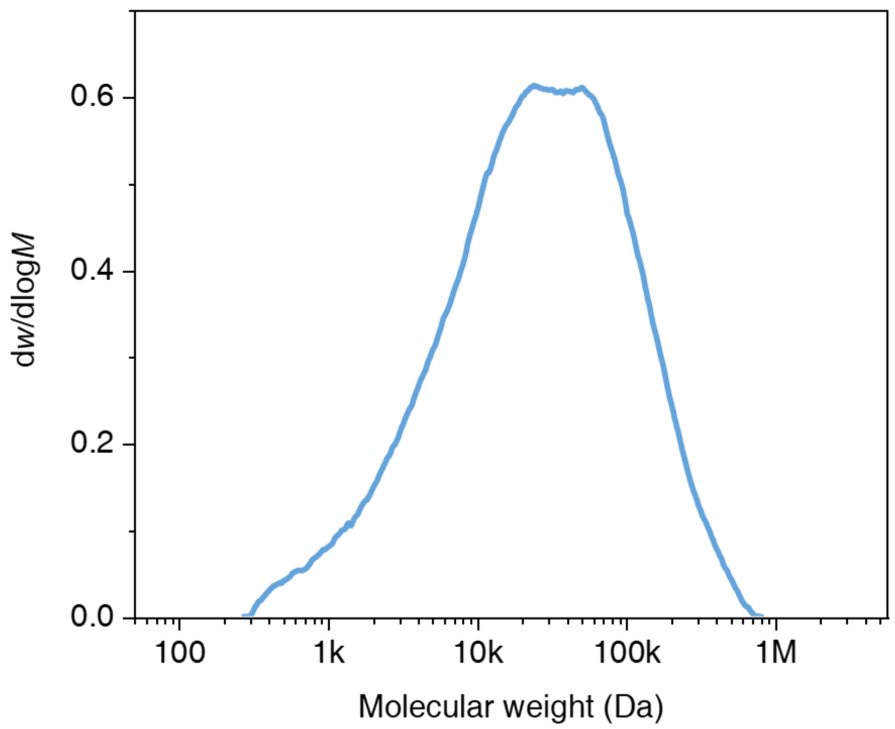

Supplementary Fig. 10 | GPC analysis of the molecular weight distribution of LDPE after photothermal treatment without any catalyst. The high molecular weight of polymer residues (M_w = 56.0 kDa, $Đ$ = 7.2) and low degradation percentage (< 1%) indicated minimal scission of C-C bonds. Reaction conditions: 180 °C (the temperature provided by 3.0 W cm^{-2} Xe lamp), 1 bar H_2/Ar (v/v = 30/70), 100 mg LDPE, reaction time 20 h.

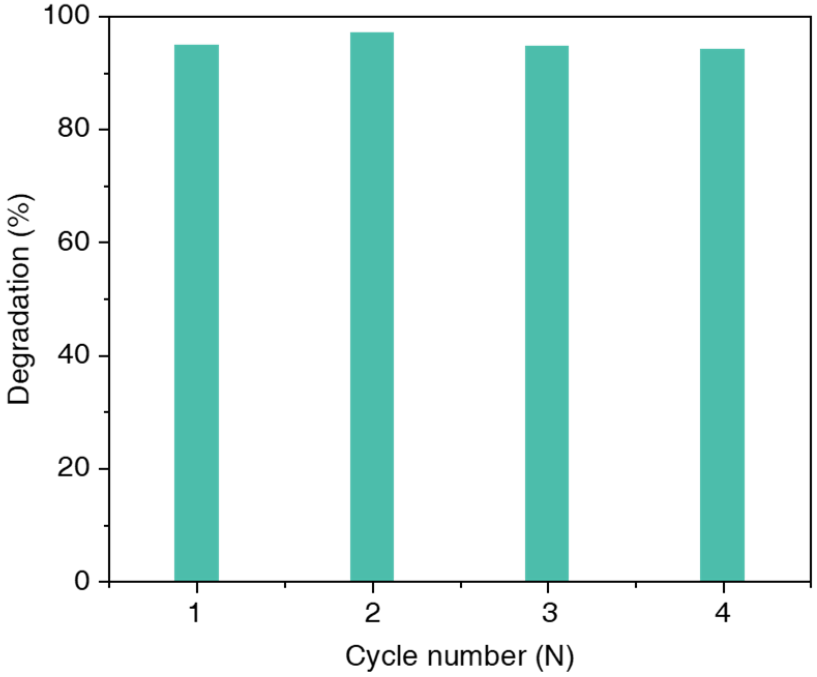

Supplementary Fig. 11 | Reusability of the Ru/TiO_2 catalyst for photothermal LDPE degradation. Reaction conditions: 300 °C, 1 bar H_2/Ar (v/v = 30/70), 80 mg LDPE, 20 mg Ru/TiO_2 (reused), reaction time 20 h for each cycle.

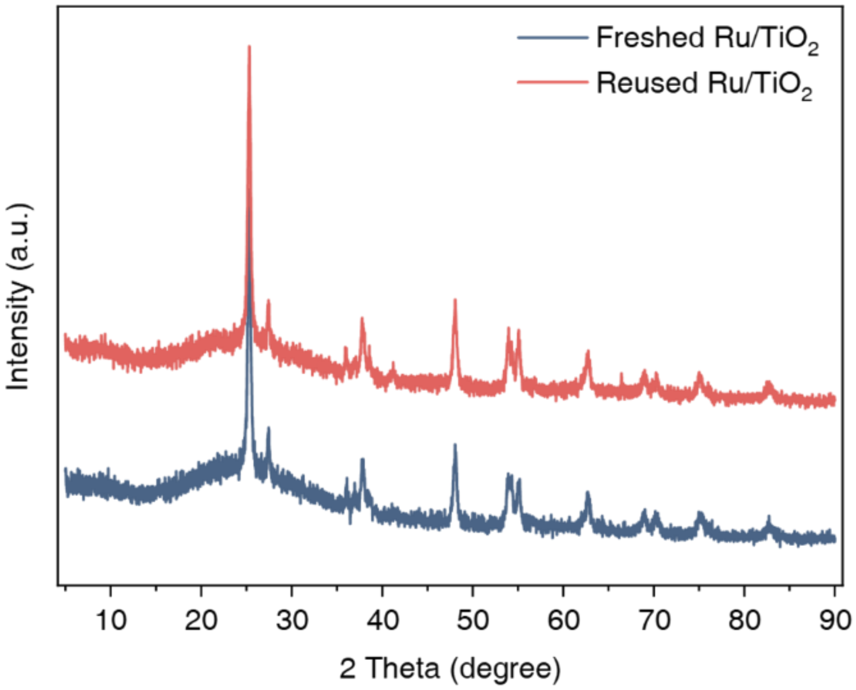

Supplementary Fig. 12 | XRD patterns of fresh Ru/TiO_2 and reused Ru/TiO_2 (4 cycles). The similarity of the XRD patterns indicates that the Ru/TiO_2 catalyst possessed excellent stability.

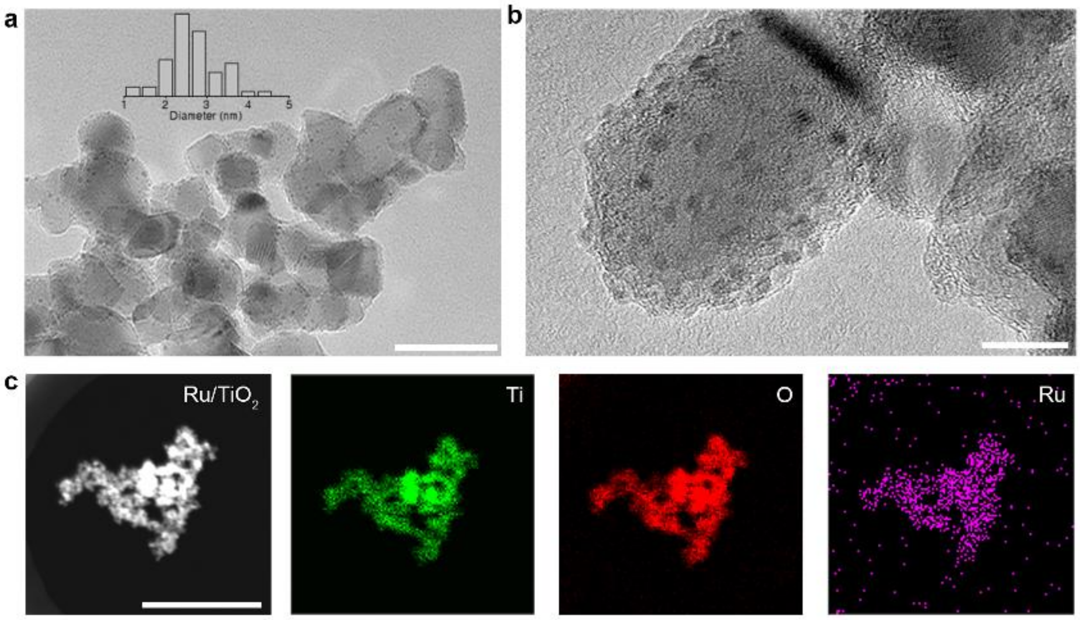

Supplementary Fig. 13 | Electron micrographs of reused Ru/TiO_2. a, TEM image of reused Ru/TiO_2 after four reaction cycles, scale bar, 50 nm. Inset: corresponding particle size distribution of Ru nanoparticles on reused Ru/TiO_2. **b**, HRTEM image of reused Ru/TiO_2 after four reaction cycles. Scale bar, 10 nm. **c**, Element maps showing the uniform distribution of Ru, Ti, and O in Ru/TiO_2. Scale bar, 300 nm.

The TEM and HRTEM images of the used Ru/TiO_2 catalyst showed Ru nanoparticles of size 2-3 nm uniformly dispersed on the TiO_2 support after the four reaction cycles. Further, the elemental distribution in the used catalyst was similar to the fresh catalyst. Results verified the excellent stability and reusability of the Ru/TiO_2 catalyst.

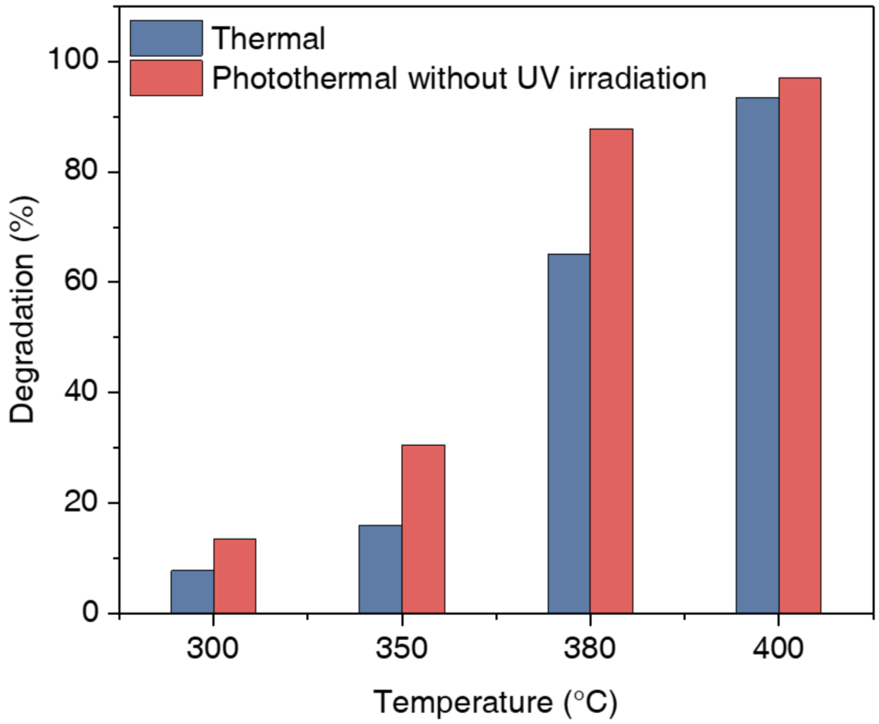

Supplementary Fig. 14 | Contribution of local heating to LDPE degradation. Degradation percentage of LDPE at different reaction temperatures under thermal degradation or photothermal degradation without UV irradiation. Reaction conditions: 1 bar H_2/Ar (v/v = 30/70), 80 mg LDPE, 20 mg Ru/TiO_2, reaction time 20 h.

When the UV light from the Xe lamp was filtered out, UV activation of LDPE could be excluded. Under such conditions, photothermal degradation of LDPE was mainly a thermal effect caused by local heating of Ru-TiO_2 under Vis and NIR light. The thermal and photothermal (without UV irradiation) LDPE degradation profiles were thus very similar in temperature.

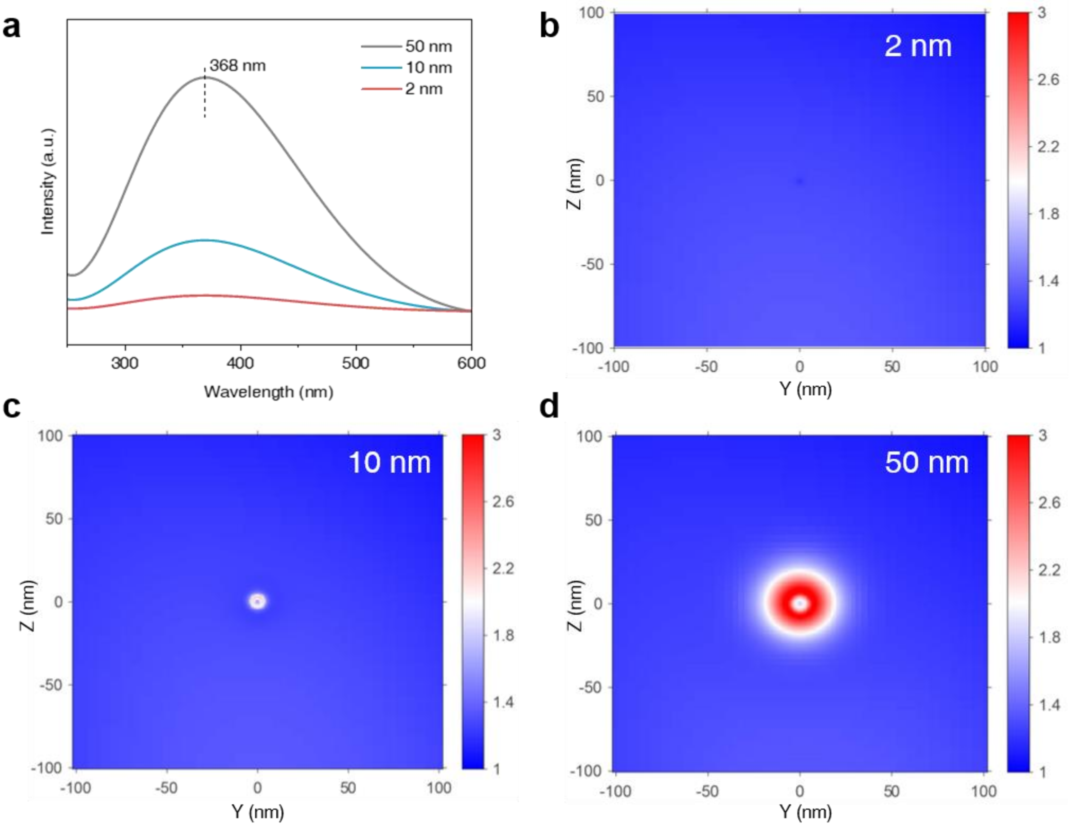

Supplementary Fig. 15 | FDTD simulation of the Ru/TiO_2 catalyst. a, Normalized LSPR absorption intensity by FDTD simulation for Ru nanoparticles with different diameters (2 nm, 10 nm, or 50 nm) loaded on a TiO_2 substrate. **b-d**, Simulated electric fields around the Ru nanoparticles supported on TiO_2. The colored bar shows the electric field intensity ($\langle|E|^2\rangle$).

The simulations show that the electric field around the Ru nanoparticles was maximized at 368 nm, close to the 310 nm reported in Moreno's work[3] (the difference in the maximum position between our work and Moreno's work is due to the different calculated parameters and nanoparticle size). The absorption intensities of the LSPR of Ru nanoparticles decreased as the nanoparticle size decreased, almost disappearing when the nanoparticle diameter was 2 nm (*i.e.* similar to the ~2.5 nm in our Ru/TiO_2 catalyst).

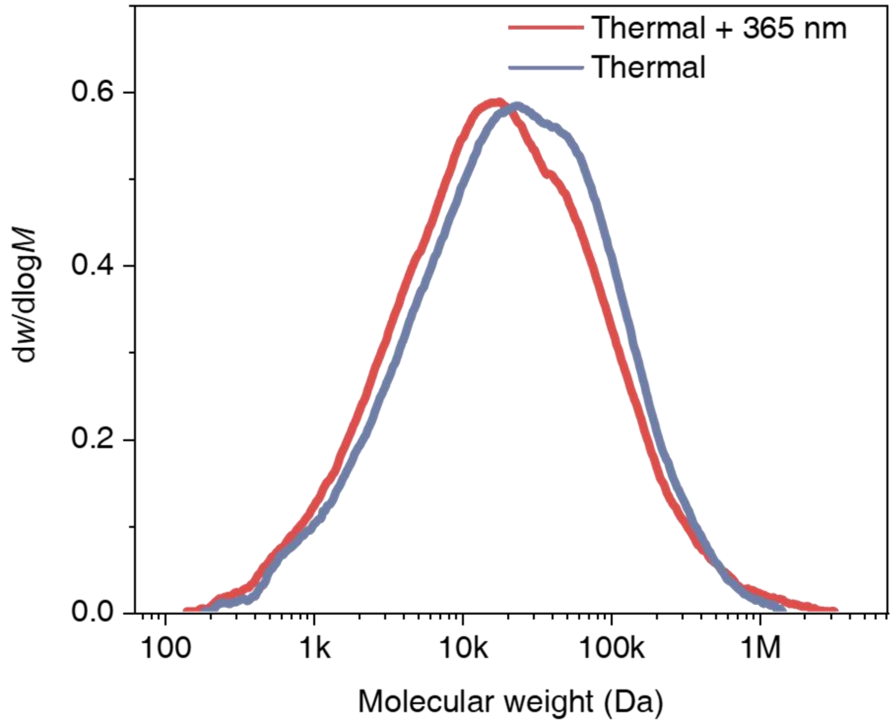

Supplementary Fig. 16 | Exclusion of photocatalytic effect or LSPR effect over Ru/TiO_2. GPC analysis of molecular weight distributions of polymeric residues after thermal degradation (no 365 nm irradiation) or thermal degradation + 365 nm irradiation. For thermal degradation + 365 nm, M_w = 56.0 kDa, $Đ$ = 10.4; for thermal degradation, M_w = 57.3 kDa, $Đ$ = 8.6. Reaction conditions: 300 °C, 1 bar H_2/Ar (v/v = 30/70), 80 mg LDPE, 20 mg Ru/TiO_2, 5 h reaction.

Based on the UV-Vis diffuse reflectance spectra for TiO_2 (Supplementary Fig. 1) and LDPE (Fig. 3d), 365 nm light should be able to induce photocatalytic activity in TiO_2 but will not be absorbed by LDPE. The data in Supplementary Fig. 16 exclude the possibility that a photocatalytic effect involving TiO_2 contributed to LDPE degradation since data collected under thermal and thermal + 365 nm irradiation were nearly identical. Furthermore, Ru nanoparticles show a maximum intensity of the generated electric field at 368 nm, very close to the excitation wavelength of 365 nm. Since the molecular weight distributions in the above plot were similar under the thermal and thermal + 365 nm regimes, any promotion due to LSPR effect involving the Ru nanoparticles was negligible.

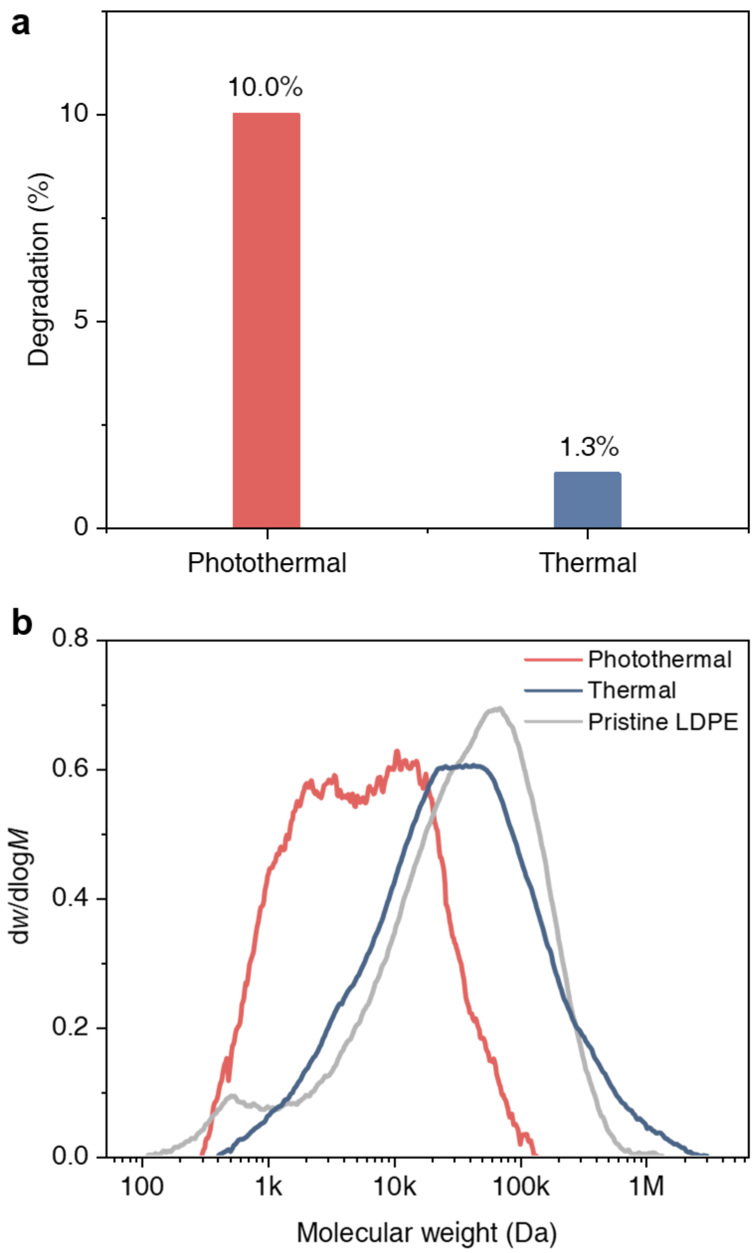

Supplementary Fig. 17 | Direct irradiation of LDPE without any catalyst. a, Degradation percentage and **b**, GPC molecular weight distributions of pristine LDPE or LDPE after photothermal or thermal degradation experiments without any catalyst. Reaction conditions: 300 °C provided by Xe lamp at 3.00 W cm^{-2} and auxiliary heating (photothermal), or only heating in the dark (thermal), 1 bar H_2/Ar (v/v = 30/70), 100 mg LDPE, reaction time 20 h.

For the photothermally treated sample, M_w = 11.4 kDa and $Đ$ = 4.3. For the thermally treated sample, M_w = 92.0 kDa and $Đ$ = 8.7. The thermally treated LDPE had a higher molecular weight than the pristine LDPE (M_w = 68.7 kDa and $Đ$ = 11.4), which is attributed to crosslinking reactions during the polymer melting process[4].

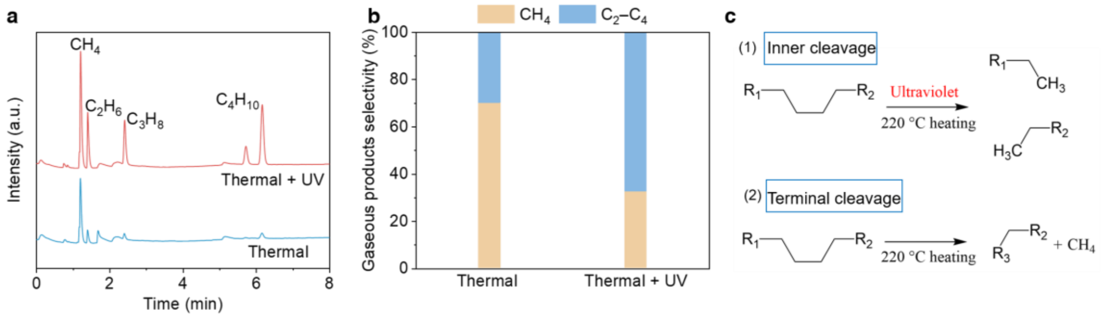

Supplementary Fig. 18 | Effects of UV irradiation on LDPE. a, FID signals of the gas chromatography. **b**, Selectivity to gaseous products after thermal degradation of LDPE in a quartz lining without any catalyst under UV irradiation (Thermal + UV) or no UV irradiation (Thermal). Reaction conditions: 220 °C, 20 bar H_2/N_2 (v/v = 70/30), 700 mg LDPE, reaction time 3 h. **c**, Proposed LDPE cleavage modes with or without UV irradiation.

As shown in Supplementary Fig. 18, CH_4 production was greatly suppressed after the introduction of UV light. Previous studies of LDPE thermal decomposition have shown that CH_4 generation results from direct terminal C-C scission (methane produced *via* a surface cascade of consecutive C-C scissions can be nearly ignored without any catalyst at this reaction temperature)[5]. Lower production rates of CH_4 under UV irradiation indicated preferential internal C-C scission instead of terminal C-C scission, implying that internal C-C bonds were more easily weakened by UV irradiation, and thus changing the cleavage mode[6]. Thus, quicker degradation of LDPE and higher selectivity of liquid fuels can be achieved using the photothermal reaction.

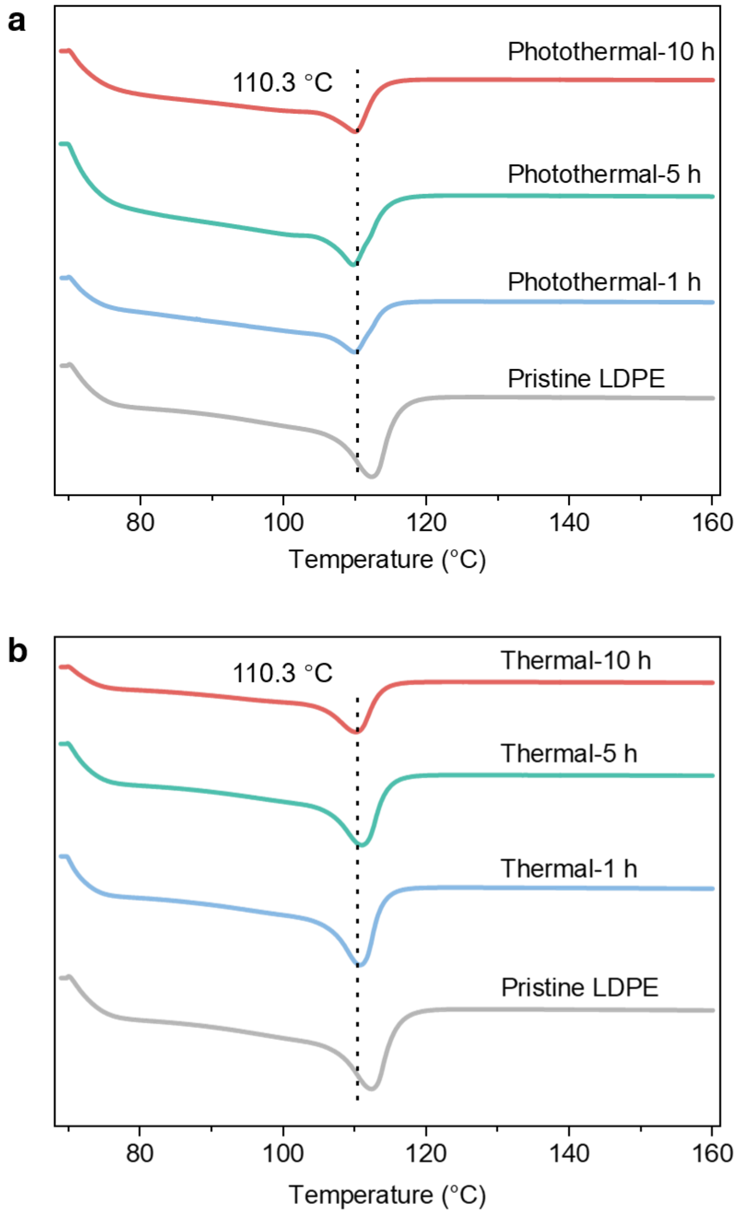

Supplementary Fig. 19 | DSC curves for LDPE following photothermal and thermal treatments with Ru/TiO_2 catalyst. DSC results for pristine LDPE and polymeric residues after 1 h, 5 h, and 10 h of reaction under **a**, photothermal degradation conditions or **b**, thermal degradation conditions. Reaction conditions: 300 °C, 1 bar H_2/Ar (v/v = 30/70), 80 mg LDPE, 20 mg Ru/TiO_2.

The melting point of LDPE residues obtained under photothermal or thermal degradation conditions stabilized at ~110 °C, slightly lower than the melting point of pristine LDPE (~112 °C). Results suggested that the photothermal and thermal degradation processes involved a similar degradation path[7].

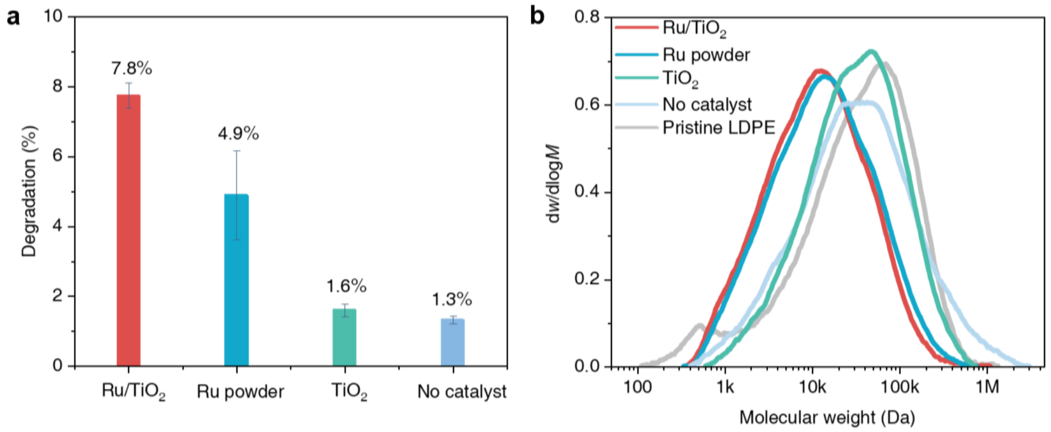

Supplementary Fig. 20 | Thermal degradation of LDPE using different catalysts. **a**, Degradation percentage and **b**, GPC analysis of molecular weight distributions of LDPE after thermal degradation with/without catalyst (Ru/TiO_2, Ru powder, TiO_2). Reaction conditions: 300 °C provided by heat element in the dark, 1 bar H_2/Ar (v/v = 30/70), 80 mg LDPE (100 mg for no-catalyst condition), 20 mg catalyst, reaction time 20 h.

Notable degradation of LDPE was observed over the Ru powder, verifying the remarkable C-C bond cleavage ability of metallic Ru sites (Supplementary Fig. 20a). The difference in performance between the Ru/TiO_2 catalyst and Ru powder is ascribed to their different H_2 adsorption ability and activation properties arising from the distinct specific surface area and Ru nanoparticle size. The action of Ru sites in promoting C-C bond cleavage during polyolefin degradation has previously been reported in studies using Ru/C[8], Ru-modified zeolite[9], and Ru/CeO_2[10] catalysts. TiO_2 showed poor performance for LDPE degradation at hydrogen partial pressures of 1 bar H_2/Ar (v/v = 30/70)[7]. As shown in Supplementary Fig. 20b, the molecular weight of the LDPE thermally treated without catalyst increased compared with pristine LDPE (M_w = 92.0 kDa and $Đ$ = 8.7 with no catalyst versus M_w = 68.7 kDa and $Đ$ = 11.4 for pristine LDPE). In the presence of the catalyst, especially Ru-containing catalysts, the molecular weights of the LDPE residues were reduced (M_w = 25.0 kDa and $Đ$ = 4.9 for Ru/TiO_2; M_w = 29.9 kDa and $Đ$ = 5.2 for Ru powder; M_w = 60.8 kDa and $Đ$ = 4.6 for TiO_2). Results confirmed that Ru-containing catalysts accelerated LDPE degradation and produced lower molecular weight polymer residues by facilitating C-C bond scission.

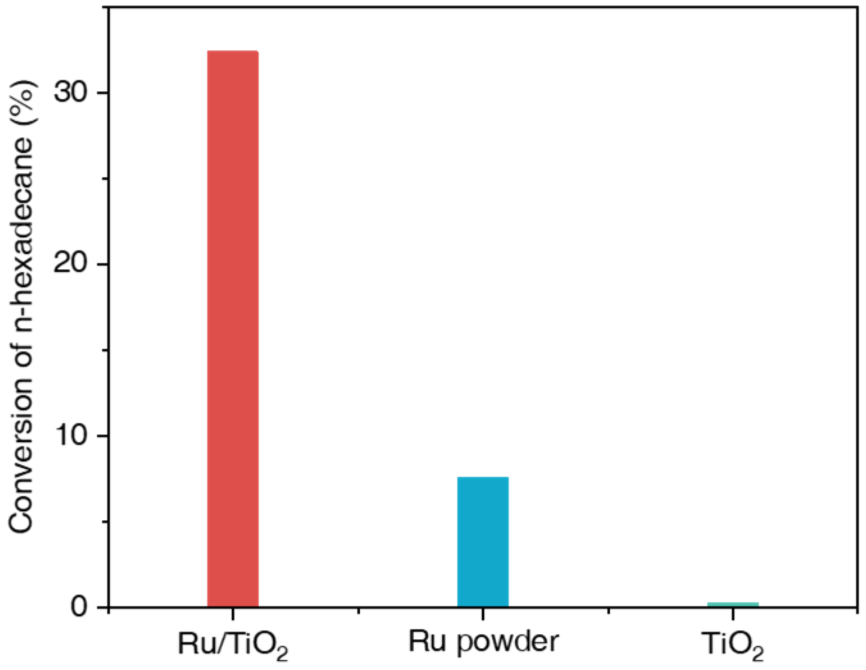

Supplementary Fig. 21 | Thermal conversion of n-hexadecane. Thermal conversion of n-hexadecane utilizing a high-pressure reactor with different catalysts (*i.e.*, Ru/TiO_2, Ru powder, TiO_2). Conversion: 32.4% for Ru/TiO_2, 7.6% for Ru powder, and 0.3% for TiO_2. This experiment adopted a similar experimental procedure with the high-pressure thermal recycling of LDPE bags. Reaction conditions: 1600 mg n-hexadecane, 100 mg catalysts, 30 bar H_2/N_2 (v/v = 70/30), 240 °C, 5 h reaction.

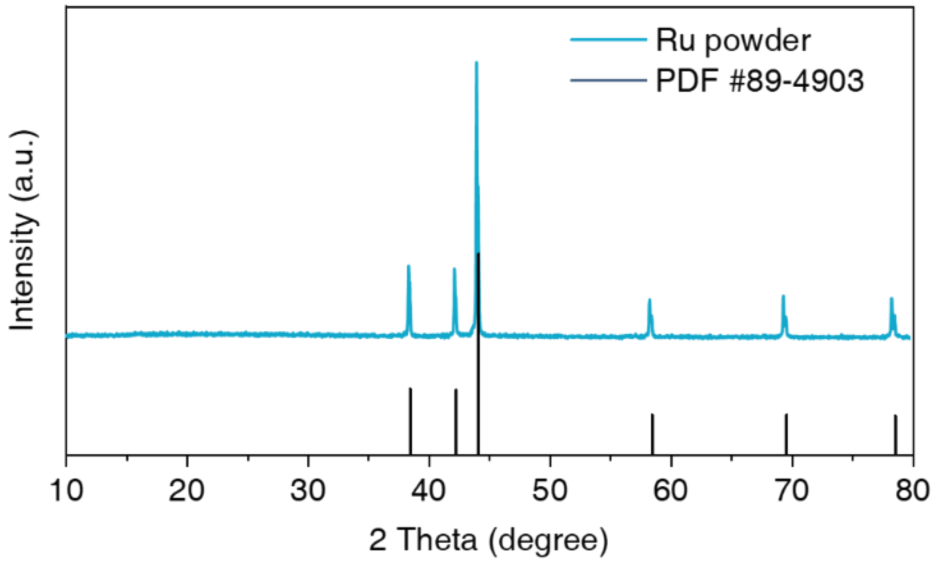

Supplementary Fig. 22 | XRD patterns of Ru powder. All peaks matched the standard card for Ru metal (PDF#89-4903).

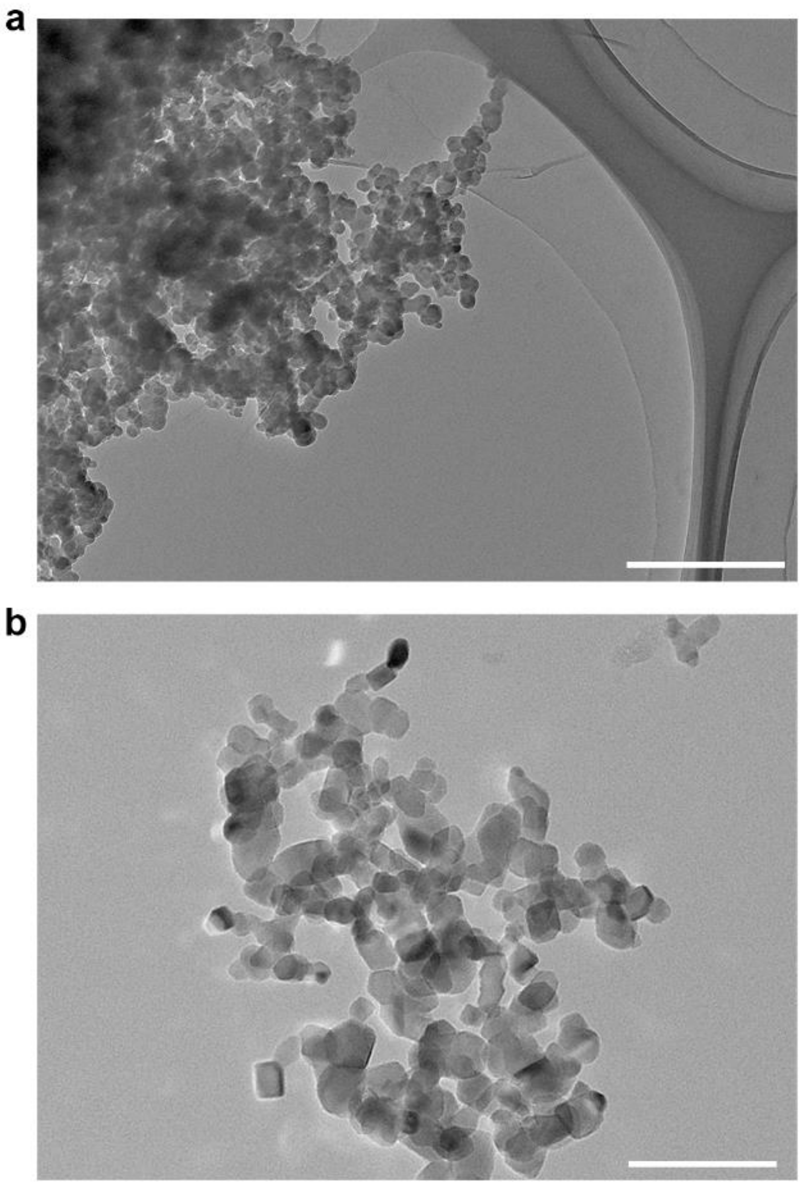

Supplementary Fig. 23 | Electron micrograph of the Ru powder and TiO_2. a, TEM image of Ru powder. Scale bar, 500 nm. **b**, TEM image of P25 TiO_2. Scale bar, 100 nm.

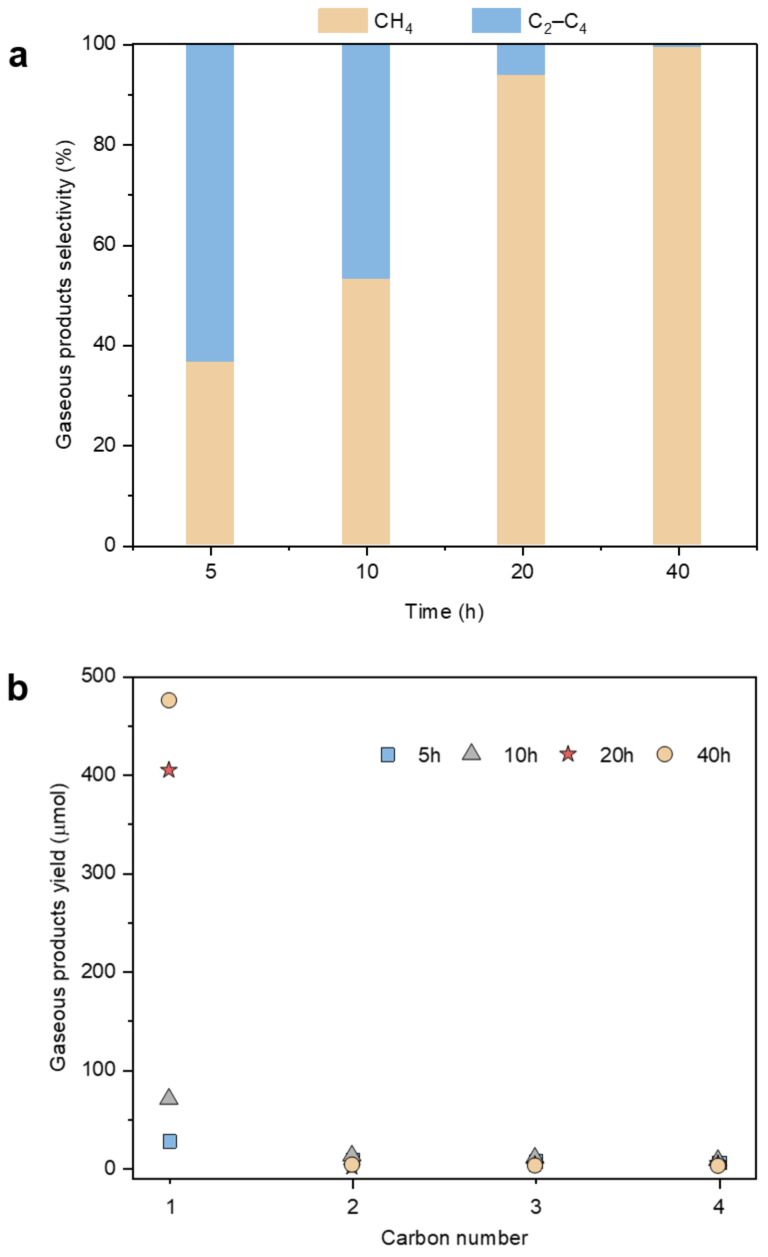

Supplementary Fig. 24 | Gaseous products of photothermal catalytic degradation of LDPE with reaction time. a, Selectivity and **b**, product yields of photothermal catalytic degradation of LDPE. Reaction conditions: 300 °C, 1 bar H_2/Ar (v/v = 30/70), 80 mg LDPE, 20 mg Ru/TiO_2.

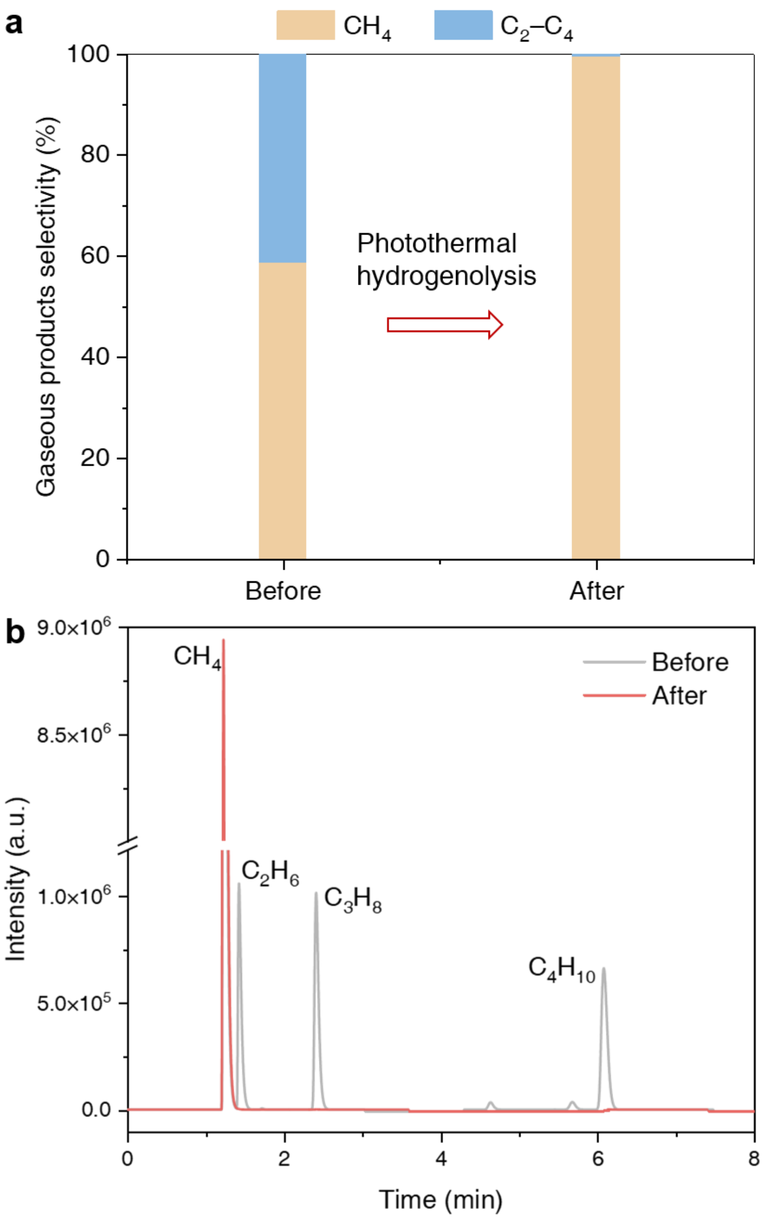

Supplementary Fig. 25 | Photothermal hydrogenolysis of light hydrocarbons over Ru/TiO_2. **a**, Selectivity of gaseous products before (left column) and after (right column) photothermal hydrogenolysis reaction. Reaction conditions: 300 °C, 1 bar H_2/Ar (v/v = 30/70), 20 mg Ru/TiO_2, reaction time 20 h. **b**, The corresponding FID signals of the C_1-C_4 products before and after photothermal hydrogenolysis of light hydrocarbons over Ru/TiO_2.

Firstly, photothermal degradation of LDPE (80 mg) in the presence of Ru/TiO_2 (20 mg) was performed at 300 °C for 10 h to obtain light hydrocarbons. Then, the light hydrocarbon products collected were injected into a reactor containing only Ru/TiO_2 for the photothermal hydrogenolysis reaction. Gaseous products were analyzed by gas chromatograph (Shimadzu, GC-2014C).

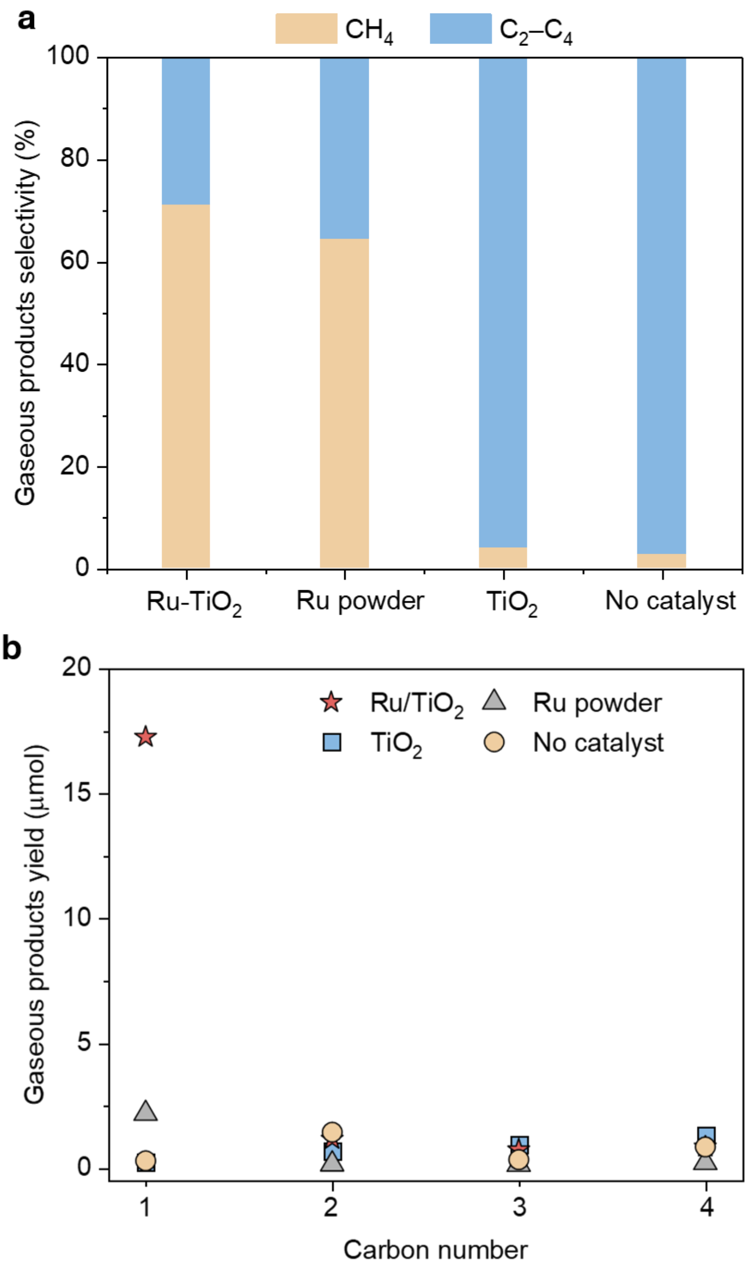

Supplementary Fig. 26 | Methanation over Ru sites. a, Selectivity and **b**, yield of gaseous products during thermal LDPE degradation over different catalysts. Reaction conditions: 300 °C, 1 bar H_2/Ar (v/v = 30/70), 80 mg LDPE (100 mg for no catalyst condition), 20 mg catalyst, reaction time 20 h.

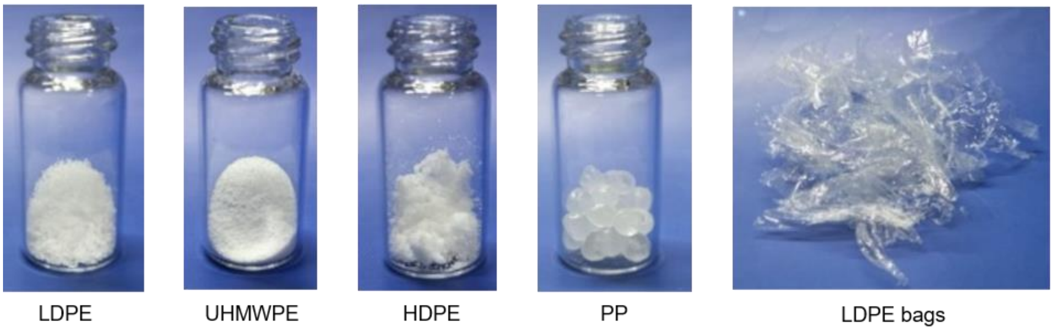

Supplementary Fig. 27 | Digital images of the different polyolefin feedstocks.

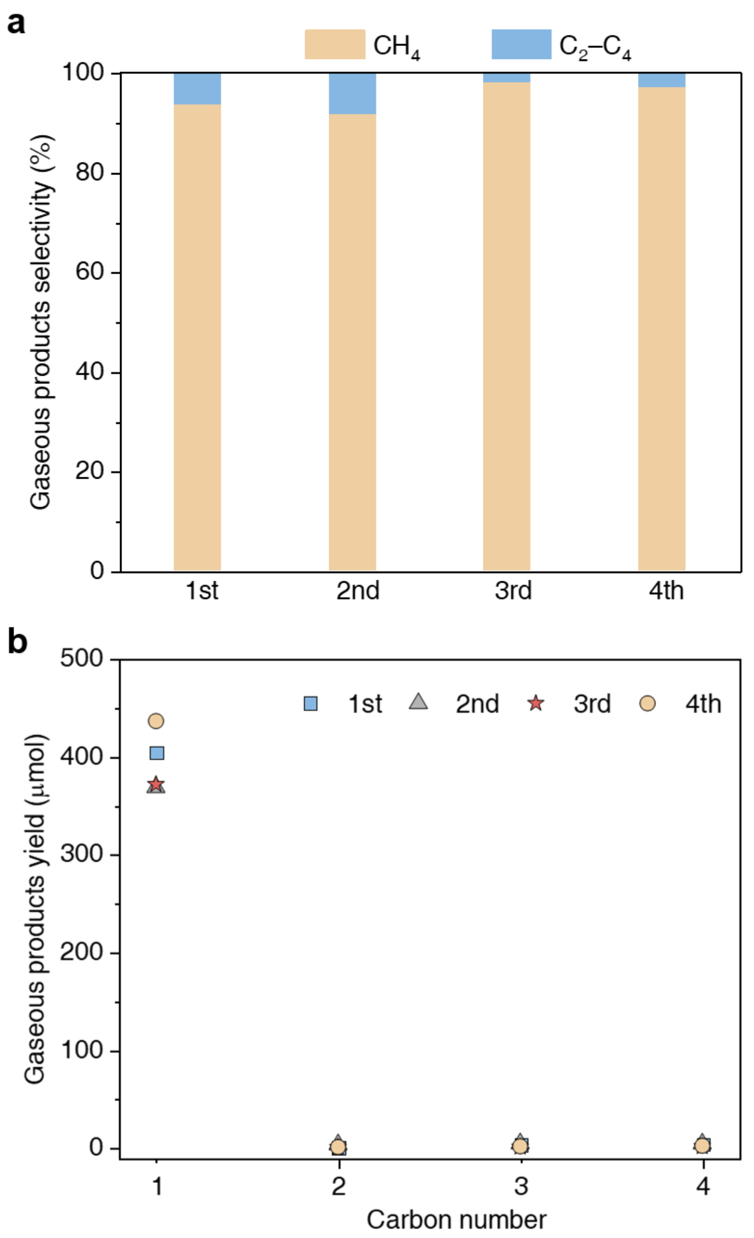

Supplementary Fig. 28 | Gaseous products of photothermal catalytic degradation of LDPE over reused Ru/TiO_2 catalyst. a, Selectivity and **b**, product yields of photothermal catalytic degradation of LDPE. Reaction conditions: 300 °C, 1 bar H_2/Ar (v/v = 30/70), 80 mg LDPE, 20 mg reused Ru/TiO_2, reaction time 20 h.

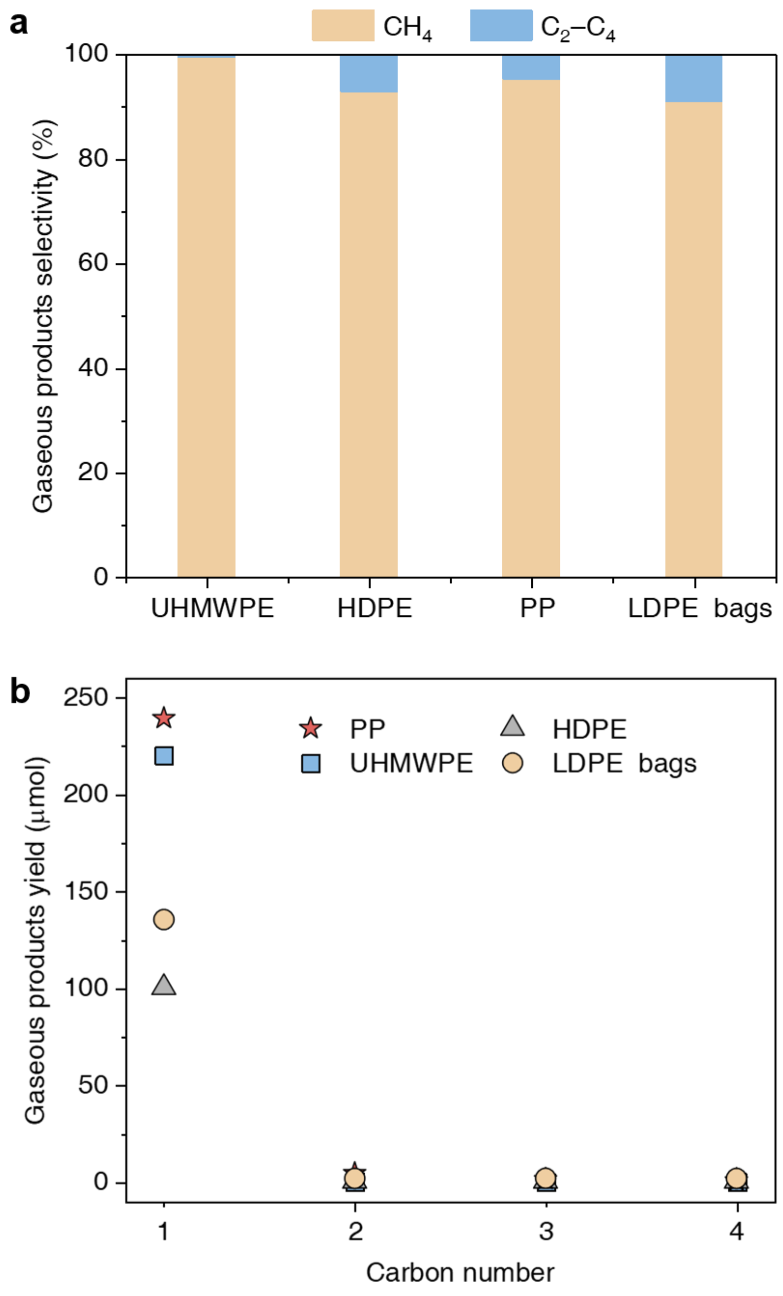

Supplementary Fig. 29 | Gaseous products of photothermal catalytic degradation. of various polyolefins. a, Selectivity and **b**, yield of gaseous products of photothermal catalytic degradation of different polyolefin feedstocks over Ru/TiO_2. Reaction conditions: 300 °C, 1 bar H_2/Ar (v/v = 30/70), 80 mg feedstock, 20 mg Ru/TiO_2, reaction time 20 h (40 h for UHMWPE).

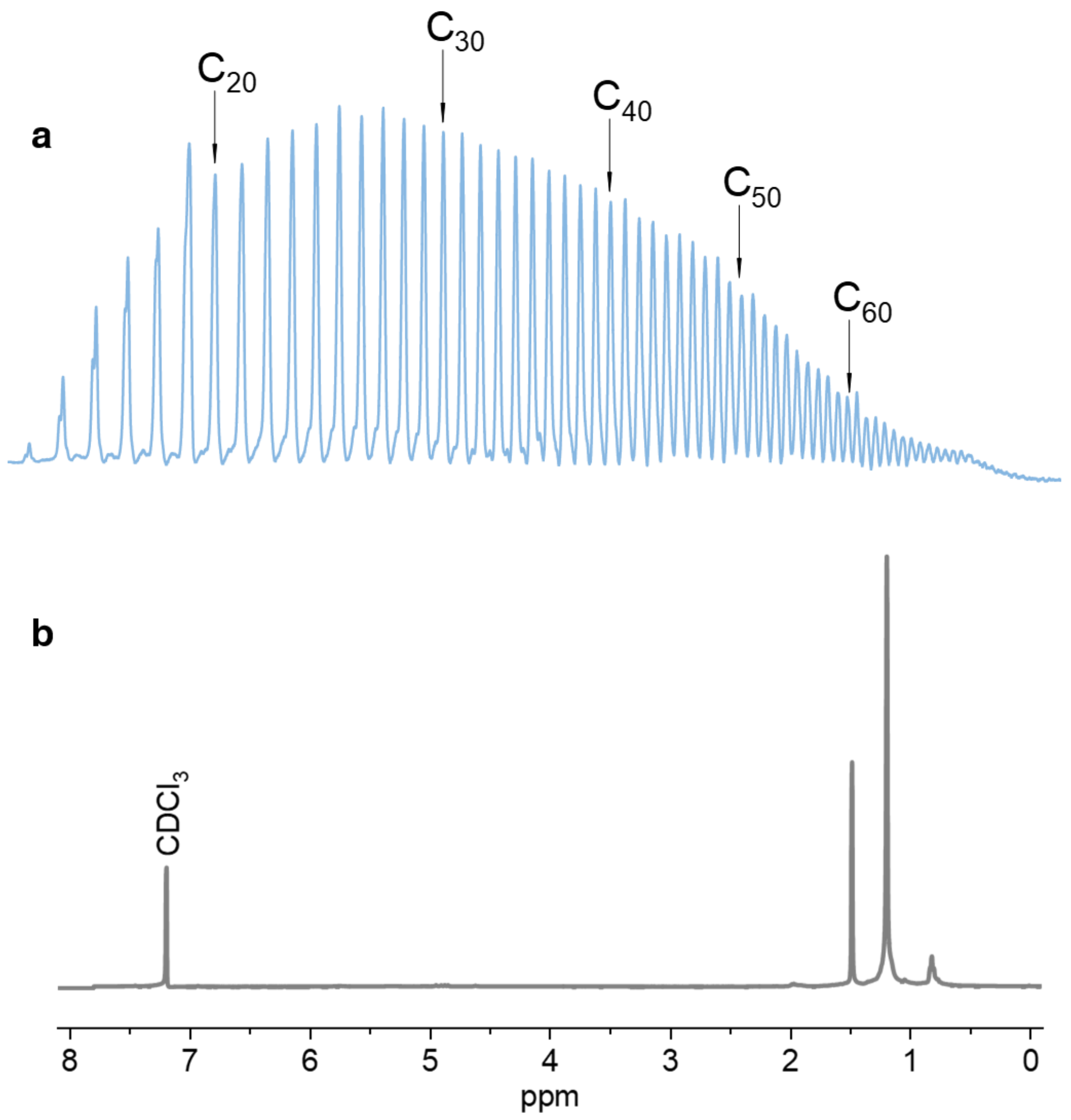

Supplementary Fig. 30 | Liquid/waxy products of photothermal catalytic degradation of LDPE. a, HTGC and **b**, ^{1}H NMR spectrum (600 MHz, 25 °C, $CDCl_3$) of the isolated liquid/waxy products of the photothermal LDPE degradation reaction. Reaction conditions: 300 °C, 1 bar H_2/Ar (v/v = 30/70), 80 mg LDPE, 20 mg Ru/TiO_2, reaction time of 20 h.

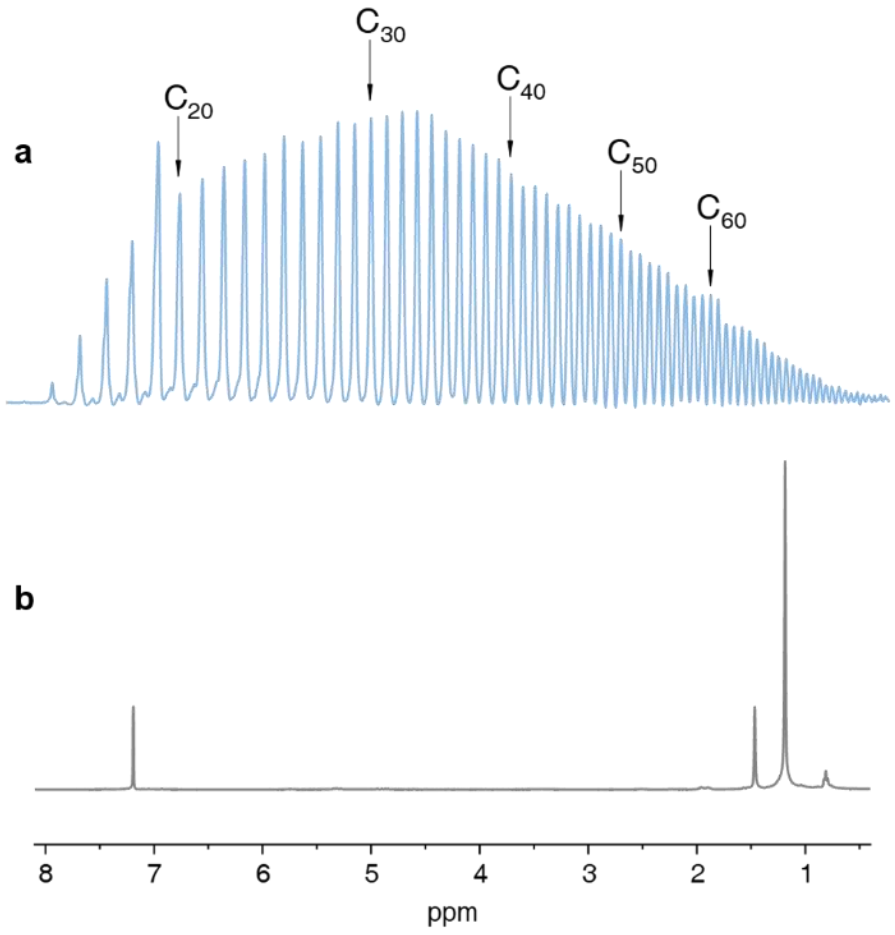

Supplementary Fig. 31 | Liquid/waxy products of photothermal catalytic degradation of UHMWPE. a, HTGC and **b**, ^{1}H NMR spectrum (600 MHz, 25 °C, $CDCl_3$) of the isolated liquid/waxy products of the photothermal UHMWPE degradation reaction. Reaction conditions: 300 °C, 1 bar H_2/Ar (v/v = 30/70), 80 mg UHMWPE, 20 mg Ru/TiO_2, reaction time 40 h.

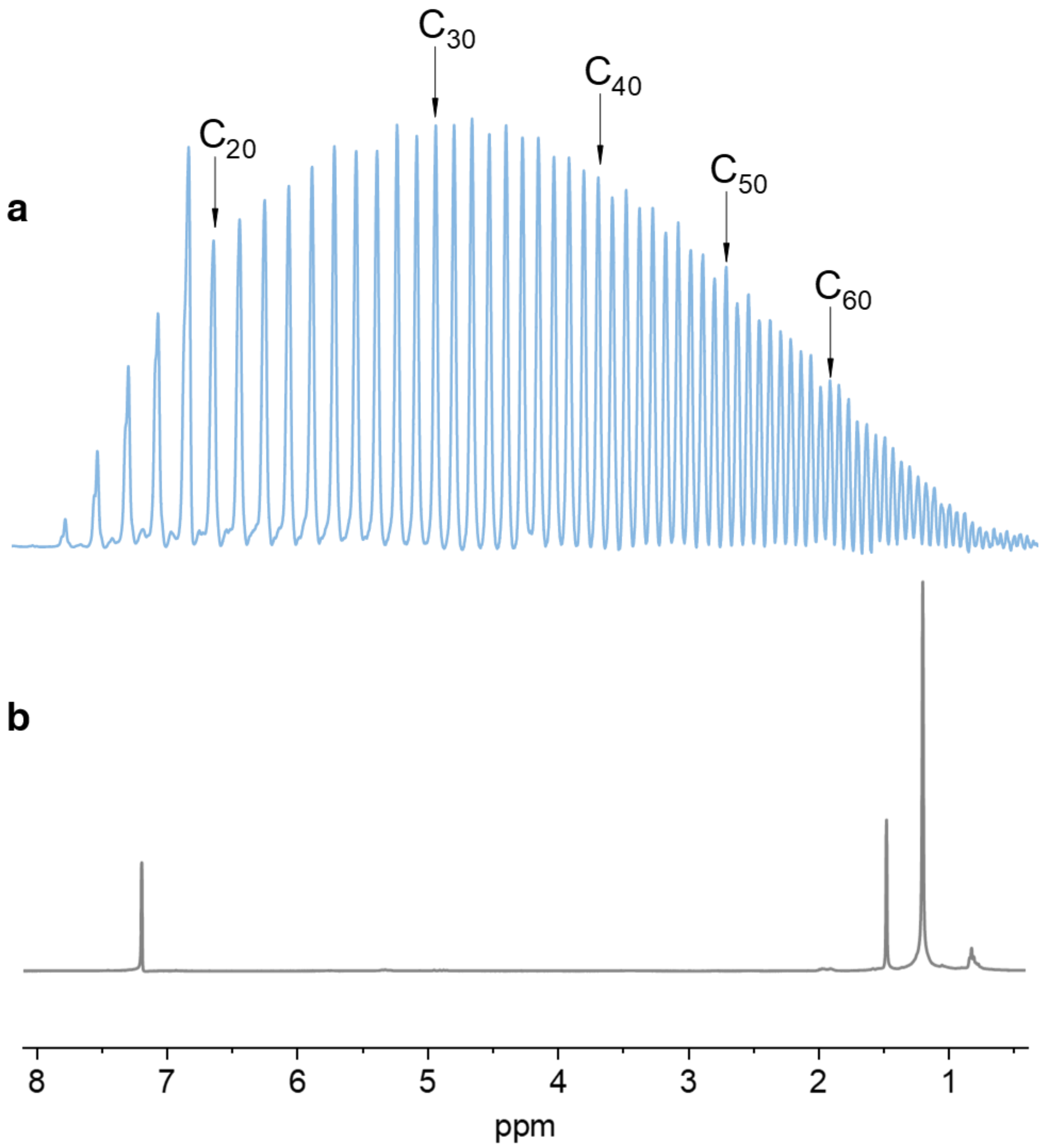

Supplementary Fig. 32 | Liquid/waxy products of photothermal catalytic degradation of HDPE. a, HTGC and **b**, ^{1}H NMR spectrum (600 MHz, 25 °C, $CDCl_3$) of the isolated liquid/waxy products of the photothermal HDPE degradation reaction. Reaction conditions: 300 °C, 1 bar H_2/Ar (v/v = 30/70), 80 mg HDPE, 20 mg Ru/TiO_2, reaction time 20 h.

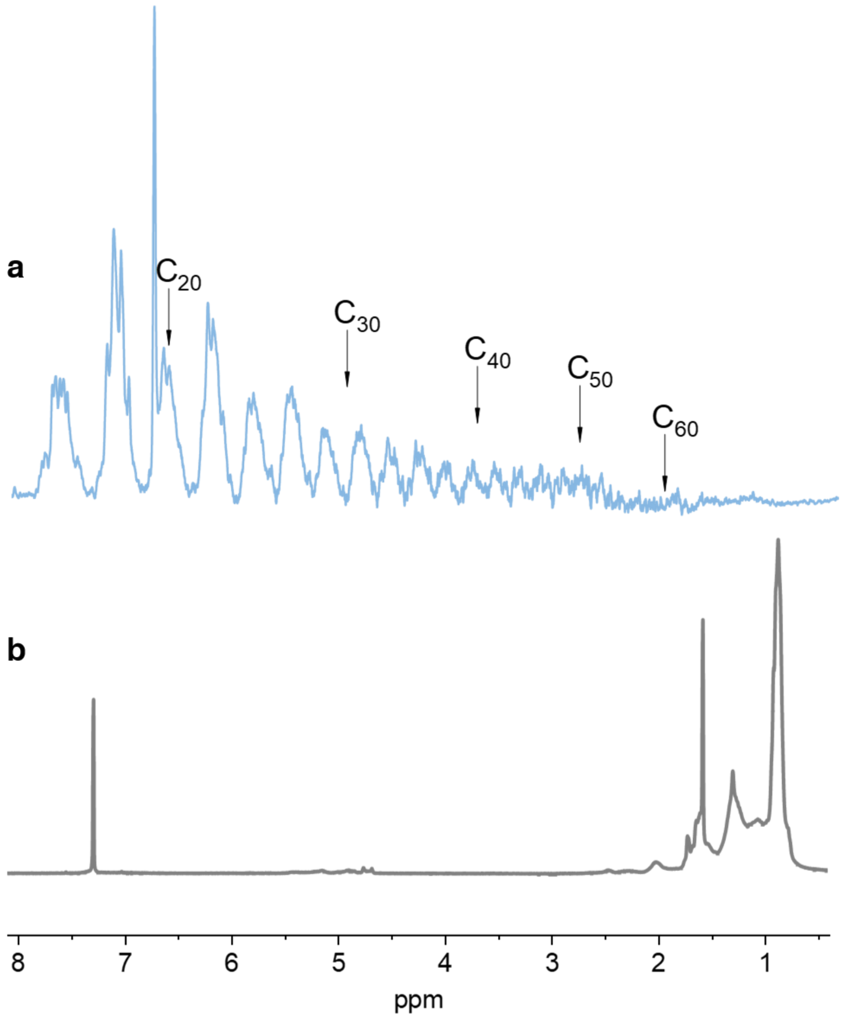

Supplementary Fig. 33 | Liquid/waxy products of photothermal catalytic degradation of PP. a, HTGC and **b**, ^{1}H NMR spectrum (600 MHz, 25 °C, $CDCl_3$) of the isolated liquid/waxy products of the photothermal polypropylene (PP) degradation reaction. Reaction conditions: 300 °C, 1 bar H_2/Ar (v/v = 30/70), 80 mg pulverized PP, 20 mg Ru/TiO_2, reaction time 20 h.

For PP, a wide range of products formed due to the polymers branched structure. A weak ^{1}H NMR signal at 4.5-5.5 ppm was ascribed to the resonances of vinylidene and vinylene. These alkene products were likely formed by mid-chain β-scission, α-chain scission followed by abstraction of a secondary proton, or disproportionation via a secondary radical[11,12].

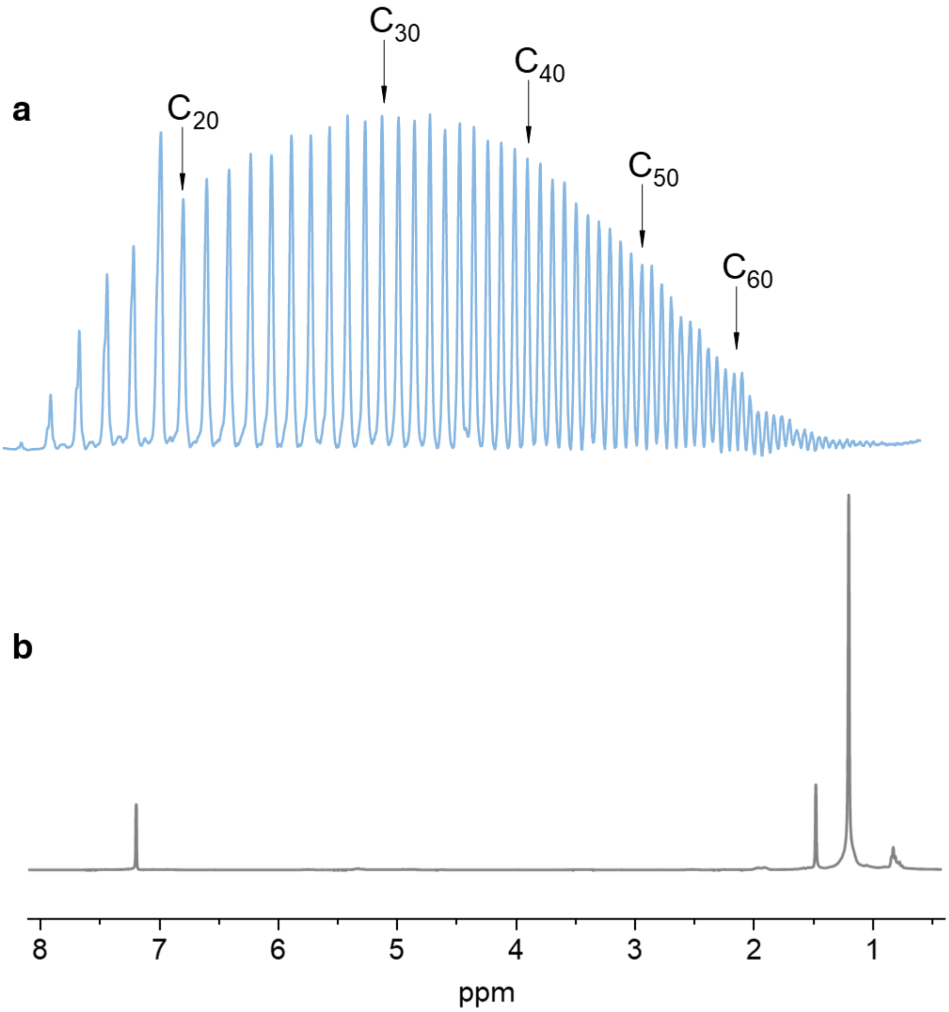

Supplementary Fig. 34 | Liquid/waxy products of photothermal catalytic degradation of LDPE bags. a, HTGC and **b**, ^{1}H NMR spectrum (600 MHz, 25 °C, $CDCl_3$) of the isolated liquid/waxy products of the photothermal LDPE bags degradation reaction. Reaction conditions: 300 °C, 1 bar H_2/Ar (v/v = 30/70), 80 mg pulverized LDPE bags, 20 mg Ru/TiO_2, reaction time 20 h.

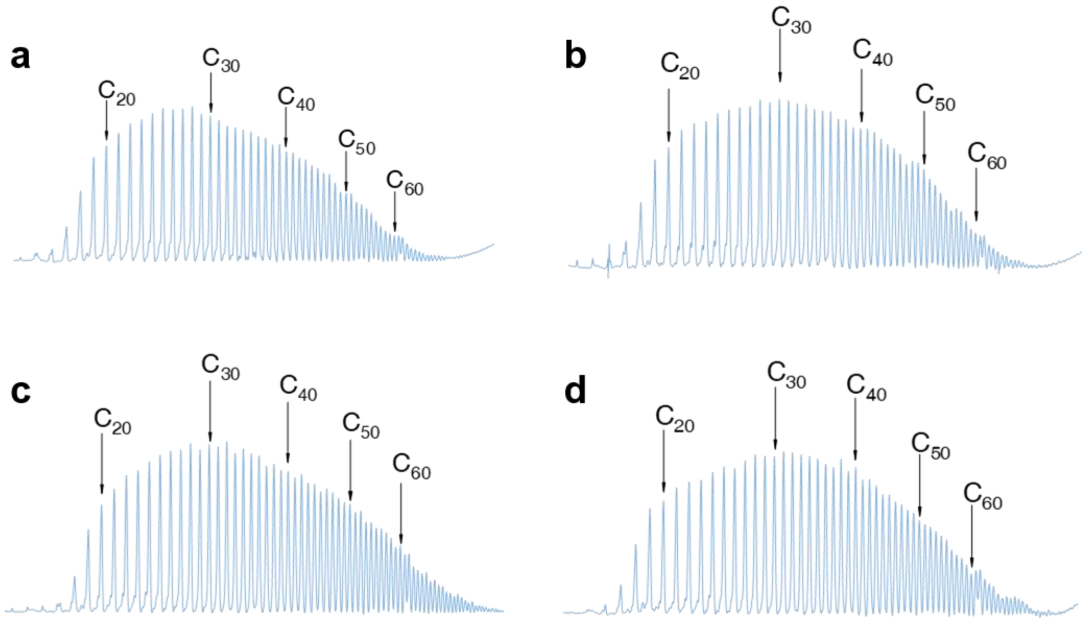

Supplementary Fig. 35 | Liquid/waxy products of photothermal catalytic degradation of LDPE over reused Ru/TiO_2 catalyst. a-d, HTGC of the isolated liquid/waxy products for the first, second, third, and fourth measurements over the reused Ru/TiO_2 catalyst, respectively. Reaction conditions: 300 °C, 1 bar H_2/Ar (v/v = 30/70), 80 mg LDPE, 20 mg reused Ru/TiO_2, reaction time 20 h.

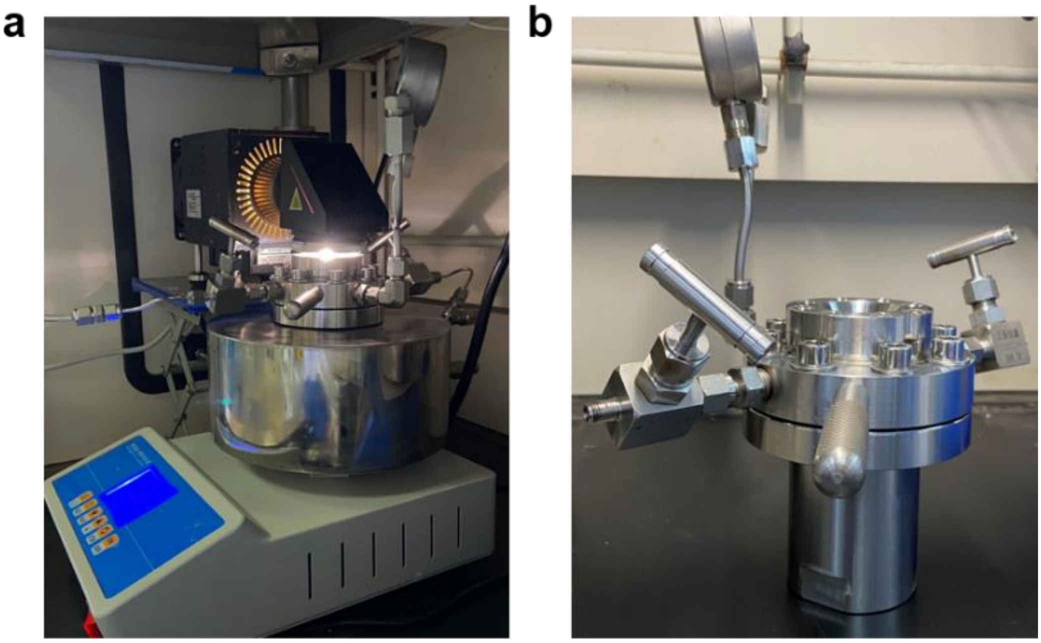

Supplementary Fig. 36 | Digital image of the high-pressure photothermal stainless reactor with sapphire window. a, The reactor during high-pressure photothermal polyolefins recycling reaction under Xe lamp irradiation. **b**, View of the high-pressure reactor.

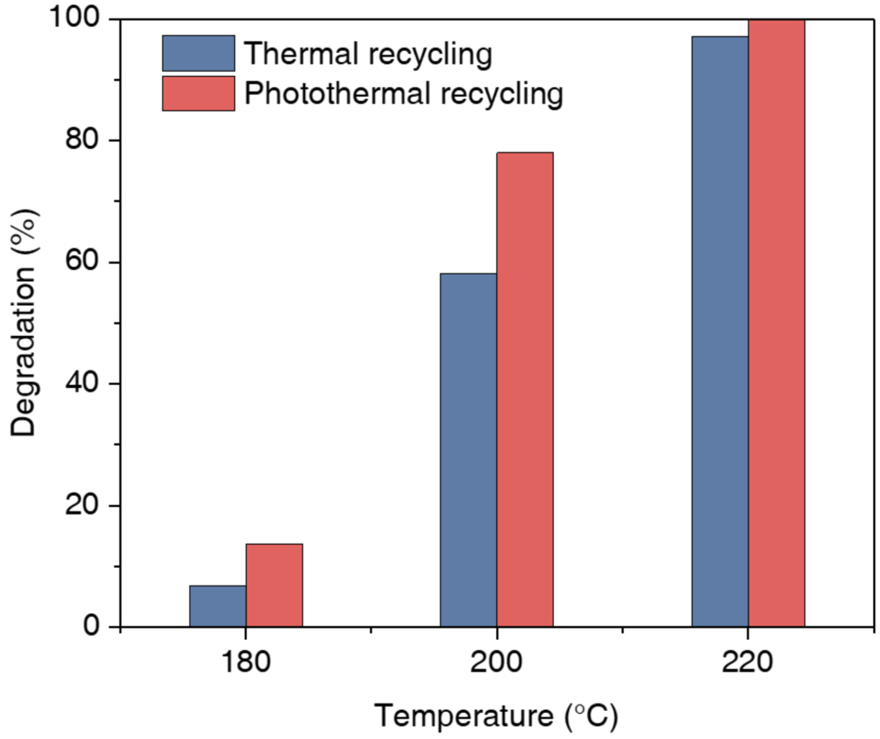

Supplementary Fig. 37 | Degradation percentage of LDPE bags under high-pressure recycling at different reaction temperatures. Reaction conditions: 180/200/220 °C under photothermal recycling (with Xe lamp irradiation) or thermal recycling (in the dark) for 3 h over Ru/TiO_2 in 30 bar H_2/N_2 (v/v = 70/30).

Benefiting from the efficient hydrogenolysis and synergistic effect of UV-Vis-NIR light, the photothermal recycling system showed enhanced LDPE recycling performance compared to thermal recycling at each temperature studied. The extent of LDPE degradation increases as the reaction temperature increased, with 100% LDPE degradation achieved during photothermal recycling at 220 °C. Results confirmed the high efficiency of the photothermal LDPE recycling system.

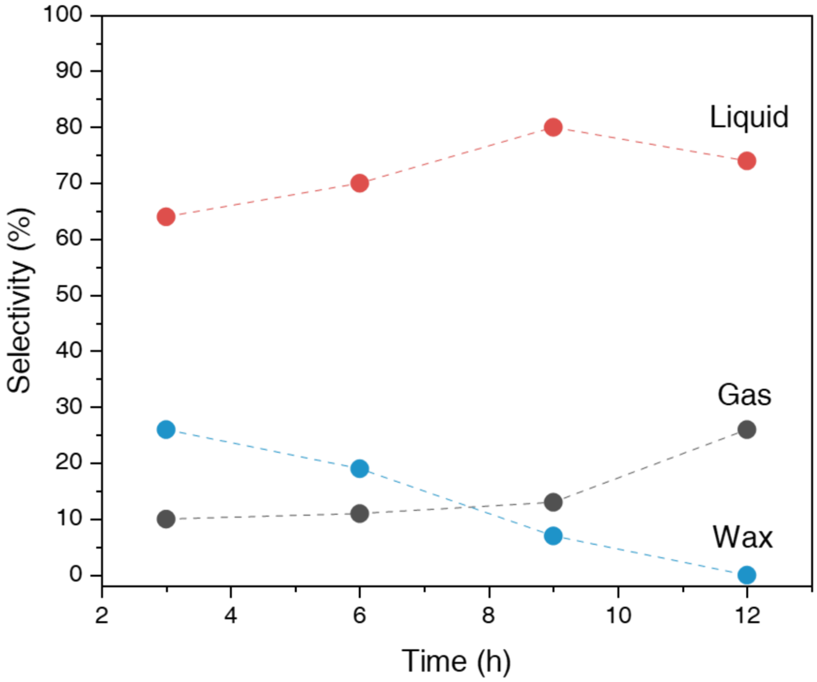

Supplementary Fig. 38 | Thermal recycling of LDPE bags at different reaction times. Product distributions of thermal recycling of LDPE bags at different reaction times. Reaction conditions: 220 °C without Xe lamp irradiation, 30 bar H_2/N_2 (v/v = 70/30), 900 mg pulverized LDPE bags, 100 mg Ru/TiO_2 catalyst.

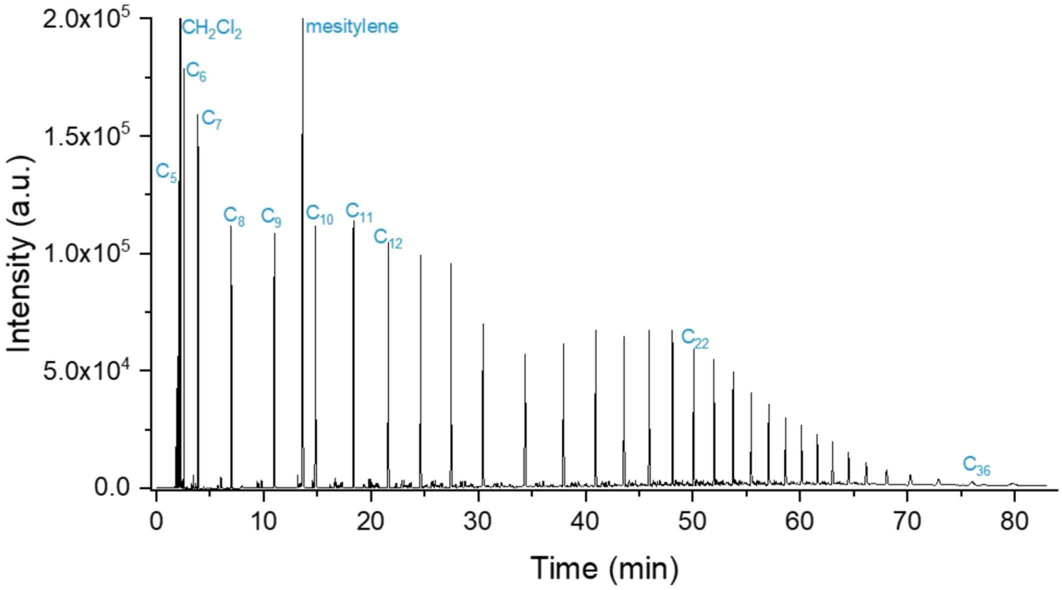

Supplementary Fig. 39 | High-pressure photothermal recycling of LDPE bags. The FID signals of the liquid/waxy products formed during the high-pressure photothermal recycling of the LDPE bags under 10 bar H_2/N_2 (v/v = 70/30) (CH_2Cl_2 was the solvent, mesitylene was the internal standard). Reaction conditions: 900 mg pulverized LDPE bags, 100 mg Ru/TiO_2 catalyst, 220 °C, reaction time 3 h.

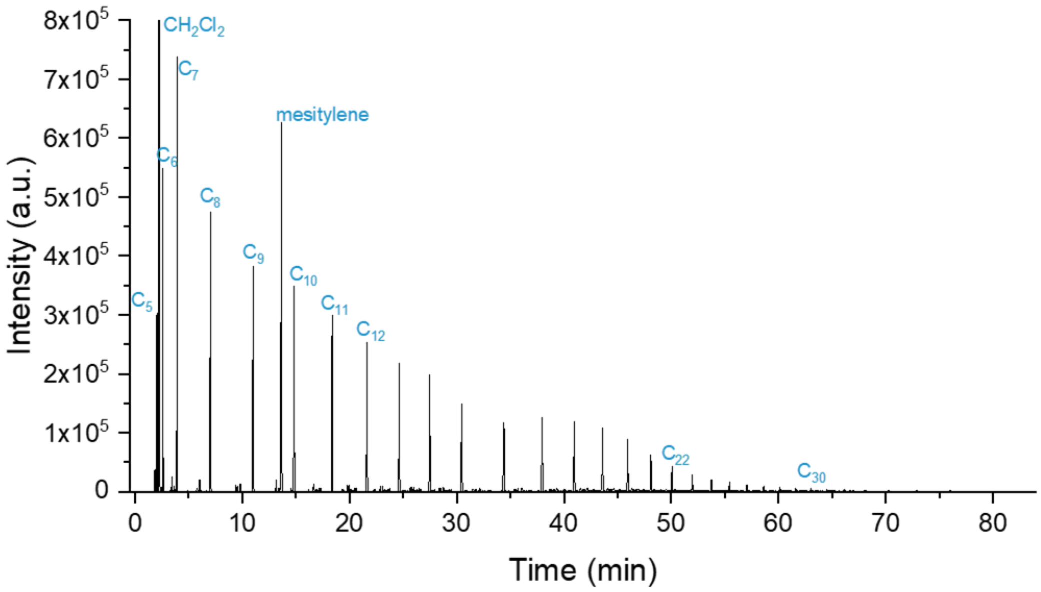

Supplementary Fig. 40 | High-pressure photothermal recycling of LDPE bags. The FID signals of the liquid/waxy products formed during the high-pressure photothermal recycling of the LDPE bags under 20 bar H_2/N_2 (v/v = 70/30) (CH_2Cl_2 was the solvent, mesitylene was the internal standard). Reaction conditions: 900 mg pulverized LDPE bags, 100 mg Ru/TiO_2 catalyst, 220 °C, reaction time 3 h.

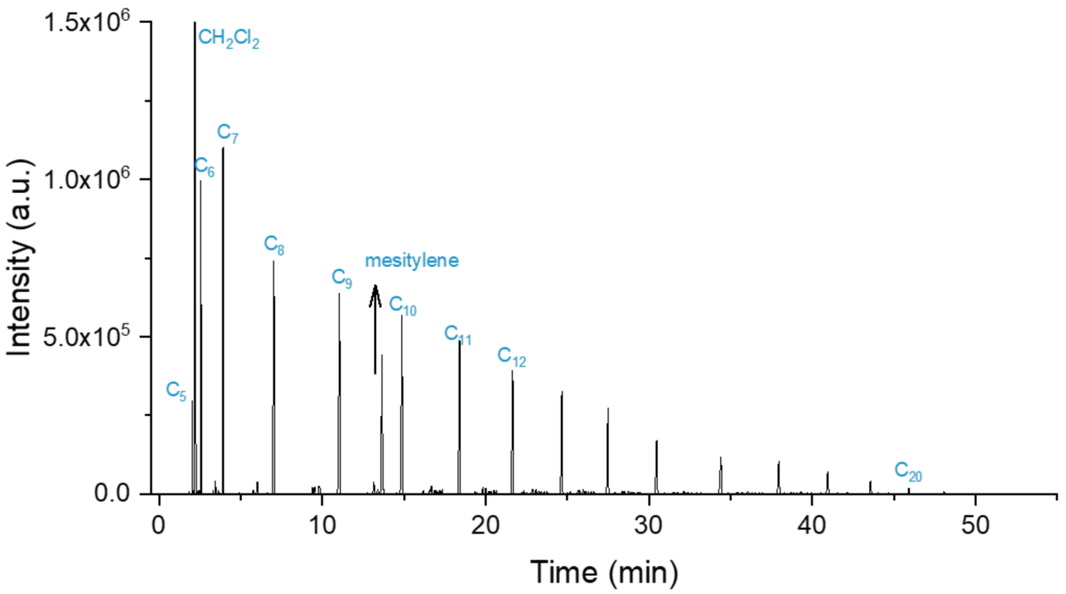

Supplementary Fig. 41 | High-pressure photothermal recycling of LDPE bags. The FID signals of the liquid/waxy products formed during the high-pressure photothermal recycling of the LDPE bags under 30 bar H_2/N_2 (v/v = 70/30) (CH_2Cl_2 was the solvent, mesitylene was the internal standard). Reaction conditions: 900 mg pulverized LDPE bags, 100 mg Ru/TiO_2 catalyst, 220 °C, reaction time 3 h.

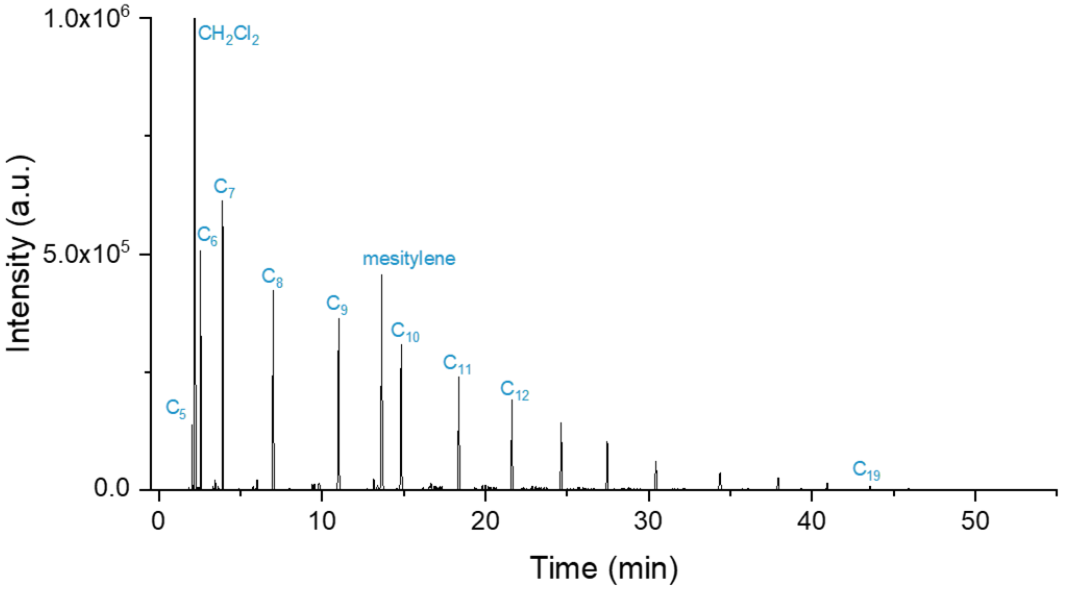

Supplementary Fig. 42 | High-pressure photothermal recycling of LDPE bags. The FID signals of the liquid/waxy products formed during the high-pressure photothermal recycling of the LDPE bags under 40 bar H_2/N_2 (v/v = 70/30) (CH_2Cl_2 was the solvent, mesitylene was the internal standard). Reaction conditions: 900 mg pulverized LDPE bags, 100 mg Ru/TiO_2 catalyst, 220 °C, reaction time 3 h.

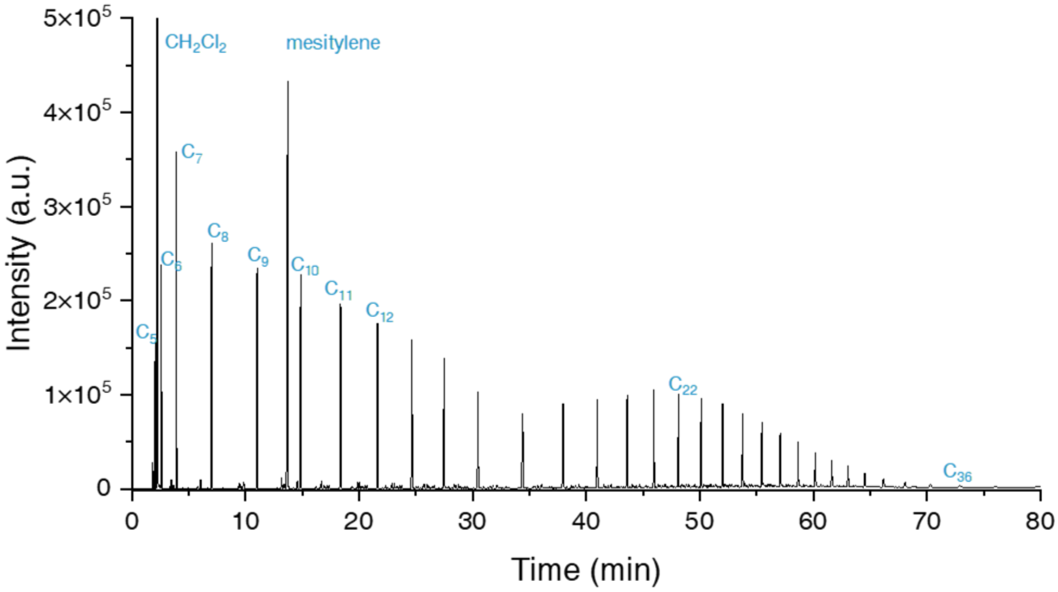

Supplementary Fig. 43 | High-pressure thermal recycling of LDPE bags. The FID signals of the liquid/waxy products formed during the high-pressure thermal recycling of the LDPE bags under 30 bar H_2/N_2 (v/v = 70/30) (CH_2Cl_2 was the solvent, mesitylene was the internal standard). Reaction conditions: 900 mg pulverized LDPE bags, 100 mg Ru/TiO_2 catalyst, 220 °C, reaction time 3 h.

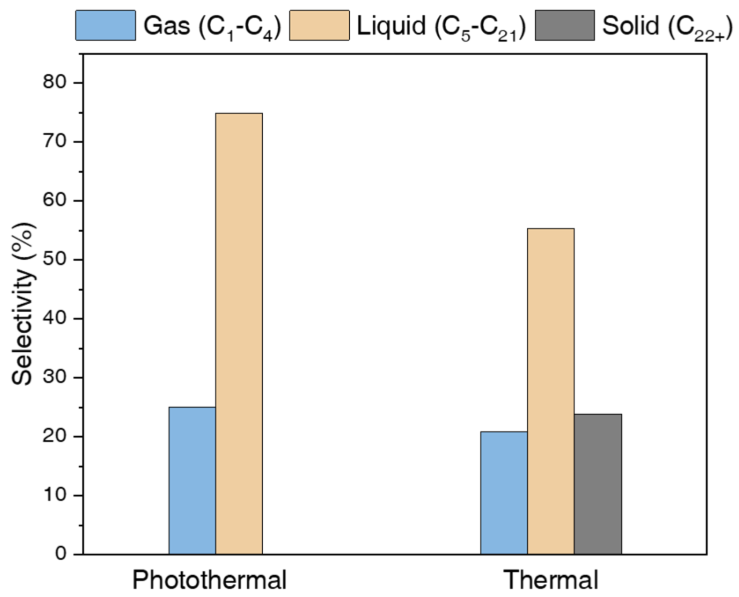

Supplementary Fig. 44 | High-pressure photothermal recycling of isotactic PP. Product selectivities of the high-pressure recycling of isotactic PP (M_w = 1.2 kDa, Sigma-Aldrich) under photothermal or thermal conditions. For thermal recycling: 21% gas, 55% liquid, 24% solid; for photothermal recycling: 25% gas, 75% liquid. Reaction conditions: 5.0 wt.% Ru/TiO_2 (50 mg), isotactic PP (2000 mg), 250 °C, reaction time 6 h, 40 bar H_2/N_2 (v/v = 70/30).

After the high-pressure photothermal recycling, no solid product remained. Further, a much higher liquid selectivity was obtained compared to thermal recycling (25% gas, 75% liquid), indicating the good universality of the photothermal recycling method.

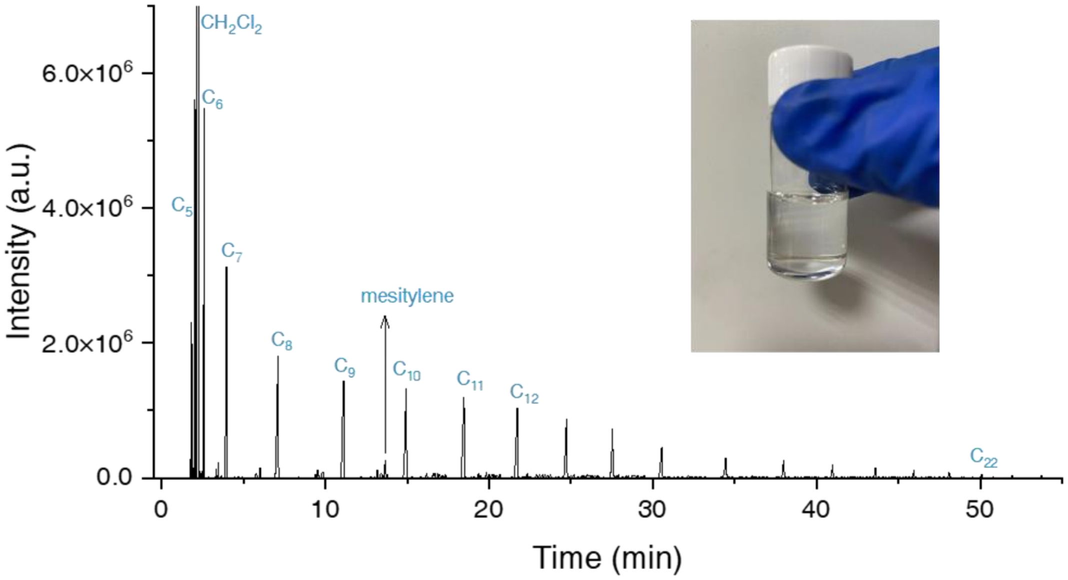

Supplementary Fig. 45 | Up-scaled high-pressure photothermal recycling of LDPE bags. The FID signals of the liquid/waxy products formed during the high-pressure photothermal recycling of the LDPE bags under 30 bar H_2/N_2 (v/v = 70/30) at 220 °C for 5 h (CH_2Cl_2 was solvent, mesitylene was the internal standard). Reaction conditions: 500 mg Ru/TiO_2, 5000 mg LDPE bags. Inset, digital photograph of the filtered liquid products.

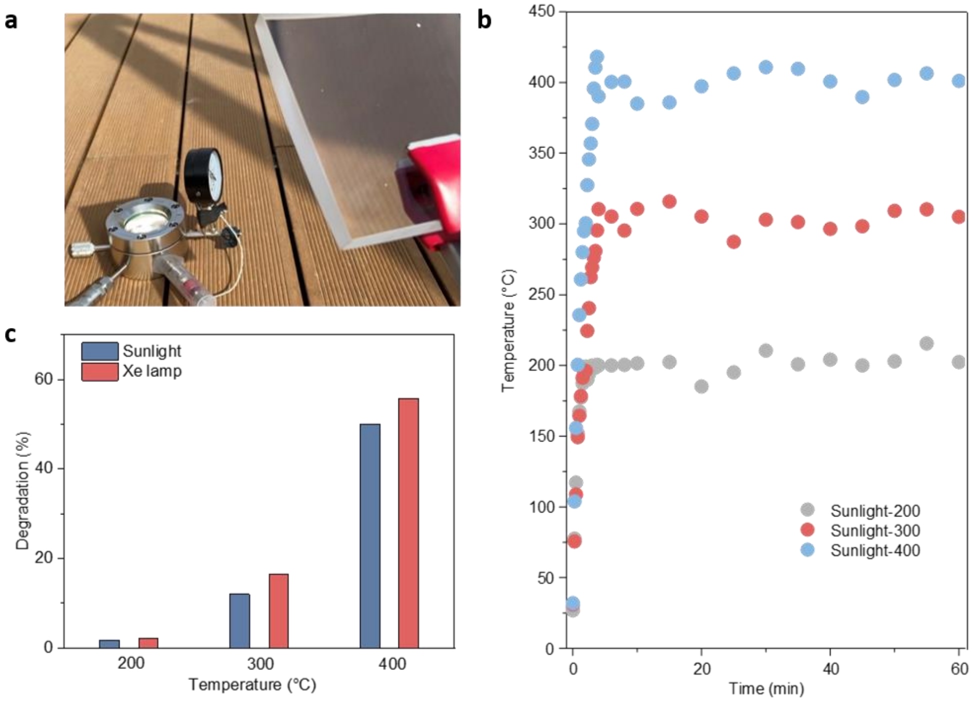

Supplementary Fig. 46 | Performance of the photothermal LDPE recycling system under concentrated sunlight. a, Photograph of reaction device with a quartz lens. **b**, Temperature profiles of the mixture of Ru/TiO_2 and LDPE under different designated photothermal reaction temperatures. **c**, Degradation of LDPE at designated temperatures under Xe lamp or concentrated sunlight. Reaction conditions: 1 bar H_2/Ar (v/v = 30/70), 40 mg LDPE, 10 mg Ru/TiO_2, reaction time 1 h. Outdoor experiments in concentrated sunlight were conducted in Beijing between 9:00 to 14:00 on 21st August 2021. This was a sunny/cloudy day with outdoor temperatures between 21-29 °C.

The LDPE degradation percentages determined under concentrated sunlight were slightly lower than those obtained using the Xe lamp due to slight fluctuations in reaction temperature and light intensity between sunny and cloudy conditions outdoors.

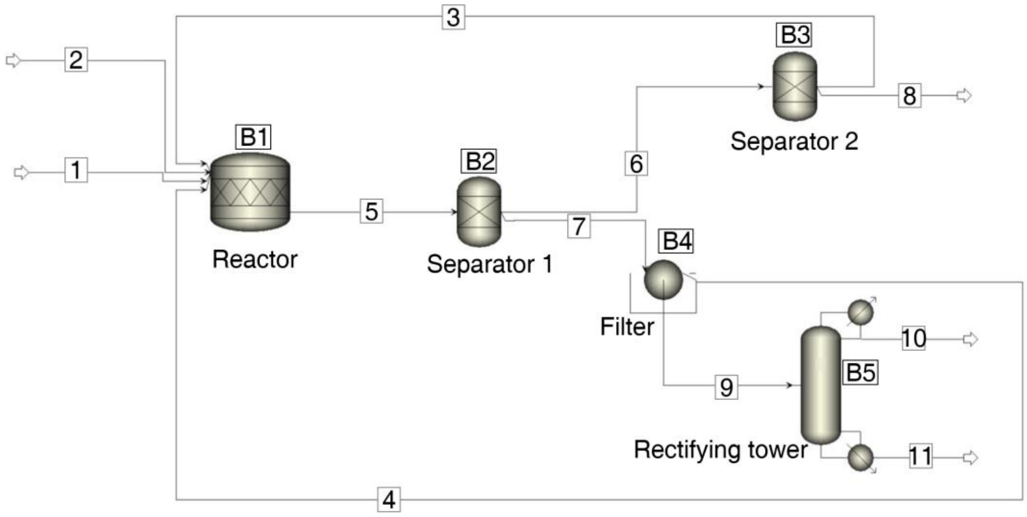

Supplementary Fig. 47 | Flow diagram of industrial polyethylene hydrogenolysis based on the simulation through Aspen Plus software.

We attempted to perform a simple technoeconomic analysis of industrial polyethylene hydrogenolysis using Aspen Plus simulation software. Here, the industrial hydrogenolysis process of polyethylene was simulated by treating 8640 tons of polyethylene per year. In a typical process, the waste polyethylene, catalyst, and H_2 are pumped into the reactor (B1) with 100% of solid conversion to produce the C_1 to C_{21} hydrocarbons, followed by the separation (B2) of gases including H_2 and C_1 to C_4 (6) and the nongaseous mixture of catalyst and C_5 to C_{21} hydrocarbons (7). Subsequently, the gas components pass through separator 2 (B3) to isolate the light hydrocarbons (C_1 to C_4) (8), with excess H_2 being recycled and fed into the reactor for the next batch of reaction (3). A solid-liquid filter (B4) was used in another product line to separate the liquid C_5 to C_{21} hydrocarbons (9), and the residual solid catalyst was recovered and reused (4). In addition, the liquids were transported to a rectifying tower (B5) to vaporize the gasoline product (C_5 to C_{12}) (10) and isolate the diesel product (C_{13} to C_{22}) (11). The energy consumed by each equipment and the total process was calculated on the basis of polyethylene hydrogenolysis by Ru/TiO_2. Compared to the two separators and the rectifying tower, the reactor consumes most of the energy (347.9 kW/h), accounting for 90.0 % of the energy input required for the whole process (Supplementary Table 3). Such energy consumption will be significantly lowered if the reactor is powered by solar energy using concentrated solar power technology[13], resulting in substantial cost reductions. Concentrated solar power technology has been reported commercially feasible in supercritical water gasification integrated with Fischer-Tropsch synthesis[14], liquid hydrocarbon fuels from CO_2 and H_2O[15], solar hydrogen production[13], liquid fuel and hydrogen coproduction[16]. In addition, molten salt thermal storage systems based on a tower design can achieve 24 h operation in the

summertime[13]. Hence, our simple analysis suggests the commercial feasibility of photothermal polyolefin recycling due to the energy savings and rapid technological development of concentrated solar power technology. The capital investment cost of building a photothermal polyolefin recycling was not considered here.

Supplementary Tables

Supplementary Table 1 | Product distributions of high-pressure photothermal recycling of LDPE bags. PD represents photothermal degradation recycling, and TD represents thermal degradation recycling. Reaction conditions: 220 °C for 3 h over Ru/TiO_2 catalyst at different reaction pressures (10, 20, 30, 40 bar H_2/N_2 (v/v = 70/30)), 900 mg pulverized LDPE bags, 100 mg Ru/TiO_2 catalyst.

LDPE bag→Gas (C_1-C_4) + liquid fuel (C_5-C_{21}) + wax (C_{22+})						
Entry	Type	Pressure (bar)	R_d (%)	Selectivity (%)		
				Gas (C_1–C_4)	Liquid fuels (C_5–C_{21})	Wax (C_{22+})*
1	PD	10	95	9	50	41
2	PD	20	100	12	80	8
3	PD	30	100	14	86	0
4	PD	40	100	17	83	0
5	TD	30	97	10	64	26

* The amount was determined by the C_{22+} products measured by gas chromatograph and the mass of low-solubility wax. R_d represents the degradation percentage.

In the photothermal recycling experiments, a substantial amount of waxy products were obtained at a pressure of 10 bar. The selectivity to liquid fuels increased gradually as the pressure increased with the highest C_5-C_{21} selectivity (86%) achieved at 30 bar. The selectivity to liquid fuels decreased with a further elevation of the reaction pressure (40 bar) due to the excessive gasification of liquid fuels. Whilst thermal recycling was capable of completely degrading the LDPE bags, photothermal recycling offered significantly higher selectivity to liquid fuels (86%) compared to thermal recycling (64%) under the same conditions. Results confirmed the superiority of the photothermal recycling system for producing liquid fuels.

Supplementary Table 2 | Product distributions of high-pressure thermal recycling of LDPE bags. TD represents thermal degradation recycling. Reaction conditions: 220 °C, 30 bar H_2/N_2 (v/v = 70/30), 900 mg pulverized LDPE bags, 100 mg Ru/TiO_2 catalyst.

LDPE bag→Gas (C_1-C_4) + liquid fuel (C_5-C_{21}) + wax (C_{22+})						
Entry	Type	Time (h)	R_d (%)	Selectivity (%)		
				Gas (C_1–C_4)	Liquid fuels (C_5–C_{21})	Wax (C_{22+})*
1	TD	3	97	10	64	26
2	TD	6	100	11	70	19
3	TD	9	100	13	80	7
4	TD	12	100	26	74	0

* The amount was determined by the C_{22+} products measured by gas chromatograph and the mass of low-solubility wax. R_d represents the degradation percentage.

Supplementary Table 3 | Energy consumption distribution of each component.

Component*	Energy consumption (kW/h)	Proportion (%)
Reactor	347.9	90.0
Separator 1	-6.5	-1.6
Rectifying tower	45.0	11.6
Total	386.4	100

* No energy consumption in separator 2 and filter.

Supplementary References

1. Truica-Marasescu, F., Jedrzejowski, P. & Wertheimer, M. R. Hydrophobic recovery of vacuum ultraviolet irradiated polyolefin surfaces. *Plasma Processes Polym.* **1**, 153–163 (2004).

2. Belmonte, G. K., Charles, G., Strumia, M. C. & Weibel, D. E. Permanent hydrophilic modification of polypropylene and poly(vinyl alcohol) films by vacuum ultraviolet radiation. *Appl. Surf. Sci.* **382**, 93–100 (2016).

3. Sanz, J. M. *et al.* Plasmon UV plasmonic behavior of various metal nanoparticles in the near- and far-field regimes: geometry and substrate effects. *J. Phys. Chem. C* **117**, 19606–19615 (2013).

4. Coates, G. W. & Getzler, Y. D. Y. L. Chemical recycling to monomer for an ideal, circular polymer economy. *Nat. Rev. Mater.* **5**, 501–516 (2020).

5. Wang, C. *et al.* A general strategy and a consolidated mechanism for low-methane hydrogenolysis of polyethylene over ruthenium. *Appl. Catal., B* **319**, 121899 (2022).

6. Vance, B. C., Kots, P. A., Wang, C., Granite, J. E. & Vlachos, D. G. Ni/SiO_2 catalysts for polyolefin deconstruction via the divergent hydrogenolysis mechanism. *Appl. Catal., B* **322**, 122138 (2023).

7. Liu, S., Kots, P. A., Vance, B. C., Danielson, A. & Vlachos, D. G. Plastic waste to fuels by hydrocracking at mild conditions. *Sci. Adv.* **7**, eabf8283 (2021).

8. Rorrer, J. E., Beckham, G. T. & Román-Leshkov, Y. Conversion of polyolefin waste to liquid alkanes with Ru-based catalysts under mild conditions. *JACS Au* **1**, 8–12 (2021).

9. Lee, W.-T. *et al.* Catalytic hydrocracking of synthetic polymers into grid-compatible gas streams. *Cell Rep. Phy. Sci.* **2**, 100332 (2021).

10. Nakaji, Y. *et al.* Low-temperature catalytic upgrading of waste polyolefinic plastics into liquid fuels and waxes. *Appl. Catal., B* **285**, 119805 (2021).

11. Kruse, T. M., Wong, H.-W. & Broadbelt, L. J. Mechanistic modeling of

polymer pyrolysis: polypropylene. *Macromolecules* **36**, 9594–9607 (2003).

12. Kolbert, A. C., Didier, J. G. & Xu, L. Mechanochemical degradation of ethylene−propylene copolymers: characterization of olefin chain ends. *Macromolecules* **29**, 8591–8598 (1996).

13. Monnerie, N., von Storch, H., Houaijia, A., Roeb, M. & Sattler, C. Hydrogen production by coupling pressurized high temperature electrolyser with solar tower technology. *Int. J. Hydrogen Energy* **42**, 13498–13509 (2017).

14. Rahbari, A., Shirazi, A., Venkataraman, M. B. & Pye, J. A solar fuel plant via supercritical water gasification integrated with Fischer–Tropsch synthesis: Steady-state modelling and techno-economic assessment. *Energy Convers. Manage.* **184**, 636–648 (2019).

15. Schäppi, R. *et al.* Drop-in fuels from sunlight and air. *Nature* **601**, 63–68 (2022).

16. He, F., Trainham, J., Parsons, G., Newman, J. S. & Li, F. A hybrid solar-redox scheme for liquid fuel and hydrogen coproduction. *Energy Environ. Sci.* **7**, 2033–2042 (2014).
